# CDX2 inhibits epithelial–mesenchymal transition in colorectal cancer by modulation of Snail expression and β-catenin stabilisation via transactivation of PTEN expression

**DOI:** 10.1038/s41416-020-01148-1

**Published:** 2020-11-26

**Authors:** Junhui Yu, Shan Li, Zhengshui Xu, Jing Guo, Xiaopeng Li, Yunhua Wu, Jianbao Zheng, Xuejun Sun

**Affiliations:** 1 Department of General Surgery, First Affiliated Hospital of Xi’an Jiaotong University, 710061 Xi’an, Shaanxi Province PR China; 2 Department of Reproductive Medicine, First Affiliated Hospital of Xi’an Jiaotong University, 710061 Xi’an, Shaanxi Province PR China

**Keywords:** Colon cancer, Metastasis, Metastasis

## Abstract

**Background:**

Emerging evidence suggests the involvement of caudal-related homoeobox transcription factor 2 (CDX2) in tumorigenesis of various cancers. Although CDX2 functions in cancer invasion and metastasis, fewer studies focus on the role of CDX2 during the induction of epithelial–mesenchymal transition (EMT) in colorectal cancer (CRC).

**Methods:**

Immunohistochemical analysis of CDX2 was performed. A series of in vitro and in vivo experiments were conducted to reveal the role of CDX2 in the invasion and metastasis of CRC.

**Results:**

CDX2 was downregulated in CRC tissues and reduced CDX2 correlated with poor prognosis. Knockdown of CDX2 promoted colon cancer cell invasion in vitro and facilitated liver metastasis in vivo with inducing EMT phenotypes. Further investigation indicated that CDX2 retarded Akt and GSK-3β phosphorylation, and thereby diminished Snail expression, β-catenin stabilisation and nuclear translocation. The depletion of β-catenin neutralised the regulation of Slug and ZEB1 by CDX2 knockdown. Mechanistically, CDX2 antagonised PI3K/Akt activity in CRC by modulating PTEN expression. CDX2 directly bound to the promoter of PTEN and transactivated its expression.

**Conclusions:**

Our study first uncovered that CDX2 inhibits EMT and metastasis of CRC by regulation of Snail expression and β-catenin stabilisation via transactivation of PTEN expression.

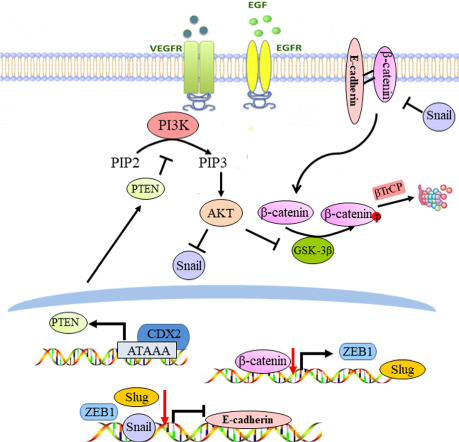

## Background

Colorectal cancer (CRC) is the second most common cause of cancer-related death.^1^ Globally, ~1,800,000 new cases are diagnosed as CRC every year. Although great progress has been achieved in early detection and multimodality treatment of CRC,^2,3^ most advanced CRC patients have a poor prognosis. Distant metastasis and relapse are the main cause of death for CRC patients.^4,5^ Emerging evidence confirmed that multiple genes and cellular pathways participate in the tumorigenesis and metastasis of CRC.^6^ Elucidating the underlying molecular pathways might highlight better therapeutic strategies for improving the prognosis of patients with CRC.

Epithelial–mesenchymal transition (EMT) is a transdifferentiation process, with the cells losing their polarity and contacts with neighbouring cells and subsequently acquiring mesenchymal-like and motile phenotypes.^7^ EMT plays pivotal and intricate roles in malignancy-related phenomena, including the cancer stem cell phenotype, drug resistance, circulating tumour cells and tumour-budding production.^8,9^ The loss of functional E-cadherin is considered as the hallmark of EMT.^10^ E-cadherin is negatively regulated by several transcriptional factors, including Snail, Slug, Twist and ZEB1/2.^11,12^

In recent years, several signalling pathways have been implicated in EMT, including the Wnt, Hedgehog, transforming growth factor-β (TGF-β), phosphoinositide 3-kinase (PI3K) and Notch pathways.^13–15^ Aberrant activation of Wnt signalling is associated with colorectal carcinogenesis.^16^ Mutations or dysregulation of the β-catenin destruction complex (APC, Axin2, CK1 and GSK-3β) results in activation of Wnt signalling.^17–19^ Phospho Akt (active Akt) inhibits GSK-3β activity by phosphorylation and subsequently decreases GSK-3β-mediated β-catenin degradation and stabilises β-catenin.^20^ An elevated nuclear β-catenin level leads to the activation of Wnt-related targets, including c-Myc, Slug, ZEB1 and MMP-7, thereby promoting cell proliferative, invasive and migratory potential.^21–23^

Caudal-related homoeobox transcription factor 2 (CDX2), an intestine-specific transcriptional factor, has been strongly implicated in the development and maintenance of intestinal mucosa.^24^ Emerging evidence supports a crucial role for CDX2 as a tumour suppressor during colorectal carcinogenesis. CDX2 expression is absent in ~30% of human CRC and is inversely associated with tumour grade.^25,26^ Mice with a CDX2^+/−^ genotype are susceptible to developing colon cancer.^27^ Our previous study indicated that CDX2 inhibited proliferation, colony formation and cell motility in CRC.^28^ Reduction of CDX2 by EGF/bFGF induces sLex/a expression by transcriptionally downregulating FUT3 during EMT induction.^29^ However, CDX2 can cooperate with β-catenin to regulate tight junctions by increasing claudin-1 expression, which promotes invasion and EMT in CRC.^30^ Therefore, the exact role of CDX2 during the induction of EMT in CRC remains controversial. In the present study, we aim to investigate the function of CDX2 in EMT and metastasis of CRC.

## Methods

### Specimens and cell culture

One-hundred-and-sixty-one CRC and paired normal colorectal (NC) tissue samples were randomly selected from CRC patients who had not received radiotherapy or chemotherapy before excision between February 2010 and September 2013. All the patients underwent surgery at the First Affiliated Hospital of Xi’an Jiaotong University. A tissue microarray of 90 pairs of primary CRC tissues was purchased from Shaanxi Kexin Biotechnology Co., Ltd. Informed consent forms were signed by all patients. Our study protocol was approved by the Ethics Committee of the First Affiliated Hospital of Xi’an Jiaotong University.

All colon cancer cells (Shanghai Institute of Cell Biology, Chinese Academy of Sciences) were maintained in RPMI-1640 medium (Gibco BRL, Carlsbad, CA, USA) supplemented with 10% foetal bovine serum (FBS) (Gibco BRL) in a humidified 5% CO_2_ atmosphere.

### Transfection

Lentiviral vectors with CDX2 shRNA or CDX2 overexpression were purchased from GeneChem Co., Ltd. (Shanghai, China). The target short-hairpin RNA (shRNA) sequences were 5′-ACAAATATCGAGTGGTGTA-3′ and 5′-GACAAATATCGAGTGGTGTAC-3′. Lentiviral infection referred to the manufacturer’s protocol.

### Liver metastasis models with colon cancer cells

The female BALB/c-nude mice (4-week-old) were purchased from a corporation of Shanghai (SLAC Laboratory Animal Co., Ltd). The mice were divided into four groups (HT-29-shCDX2/HT-29-shCtrl and SW480-shCDX2/SW480-shCtrl). The detailed protocol referred to the previous studies.^31,32^ Briefly, after anaesthesia, abdominal surgeries were conducted to expose the spleen and slowly inject it with 1 × 10^7^ cells. The spleen was then returned to the abdominal cavity, and the abdomen was closed. After 50 days of intrasplenic injection, the nude mice were anaesthetised with diethyl ether and sacrificed by cervical dislocation. All animal procedures were in accordance with the Helsinki Declaration, and approved by the Ethics Committee of The First Affiliated Hospital of Xi’an Jiaotong University.

### Wound-healing assays

The would-healing assays were carried out as described previously.^33^ Cells were cultured in six-well plates until 90% confluence. Pipette tips (10 µL) were then utilised to scratch artificial vertical lines. The cells were maintained in FBS-free medium for an additional 48 h. The images of wound closure were captured under a microscope at 0, 24 and 48 h.

### Transwell assays

The transwell assays were carried out as described previously.^33^ Briefly, cells, suspended in FBS-free medium, were seeded into Transwell (Corning, New York, NY, USA) inserts coated with Matrigel (BD Biosciences, Franklin Lakes, NJ, USA) or not. The lower chamber contained 600 μl of RPMI-1640 medium with 20% FBS. Twenty-four hours later, the migratory or invading cells were imaged and counted under an inverted microscope after crystal violet staining.

### Quantitative real-time PCR (qRT-PCR)

Total RNA extraction, complementary DNA (ctDNA) synthesis and qRT-PCR were carried out as described previously.^34^ The primers for qRT-PCR were listed in Supplementary Table 1.

### Microarray analysis

Microarray analysis was carried out to compare gene expression profile in SW480-shCDX2 and the control cells. Total RNA was extracted with TRIzol reagent and prepared for subsequent analysis by Affymetrix GeneChip system (Genechem Co., Ltd).

### Immunohistochemistry (IHC)

The IHC staining was carried out as described previously.^34^ Briefly, the extent of stained cells (0, 0–5%; 1, 6–25%; 2, 26–50%; 3, 51–75%; 4, 76–100%) and the staining intensity (0, negative; 1, light brown; 2, brown; 3, dark brown) were recorded. The immunoreactivity scores (IRSs) were defined as the product of extent and intensity scores. An IRS of >3 was considered as positive expression.

### Nuclear extract preparation and western blotting analysis

Nuclear protein was extracted using a Nuclear Extraction Kit (Abcam, Cambridge, MA, USA) as described previously.^35^ Nuclear extracts were prepared for subsequent analysis. The western blotting analysis was performed as described previously.^34^ Detailed information regarding these antibodies is shown in Supplementary Table 2.

### Luciferase reporter assay

For promoter analyses, a fragment of the PTEN 5′-flanking sequence (from –912 bp to +207 bp) and other truncated fragments were cloned into the pGL3.0 Basic Vector (Promega, Madison, WI, USA) to generate a PTEN full promoter reporter construct and the truncated ones (Supplementary Table 1). The plasmids containing firefly luciferase reporters of PTEN promoter and the truncated ones and the pTK-RL plasmids were co-transfected into cells. The detailed protocol was carried out as described previously.^34^

### Quantitative chromatin immunoprecipitation (qChIP)

The qChIP assay was conducted using the EZ-ChIP Kit (Millipore, Bedford, MA, USA) according to the method of the manufacturer’s instructions.^34^ In total, 5 μg of anti-CDX2 antibody and 1 μg of IgG-negative control antibody were used to precipitate the chromatin–protein mixture. Finally, the target fragment or endogenous non-coding region fragment were amplified with specific primers (Supplementary Table 1) by using real-time PCR.

### Immunofluorescence (IF) and immunocytochemistry (ICC)

The IF assay was carried out as described previously.^33^ The sample was observed using a fluorescence microscope to measure E-cadherin and vimentin expression and β-catenin subcellular localisation.

For ICC assay, the cells were fixed with 4% paraformaldehyde for 20 min, punched with 0.2% Triton X-100 for 10 min and then incubated with the primary antibodies. The following protocol is the same as the IHC analysis.

### Statistical analysis

The differences between the experimental and control groups were compared by the Student’s *t* test or one-way ANOVA. Correlations in the protein levels were conducted using Pearson linear-regression analysis. Survival rate was calculated using Kaplan–Meier method, and the difference in survival was analysed by log-rank test. Univariate and multivariate analyses were conducted using a Cox proportional hazard model. *P* < 0.05 was defined as statistically significant. All data were analysed using SPSS 18.0 software (SPSS Inc., Chicago, IL, USA). All in vitro experiments were carried out in triplicate.

## Results

### Low levels of CDX2 correlate with progression and poor prognosis in human CRC

**Fig. 1:**
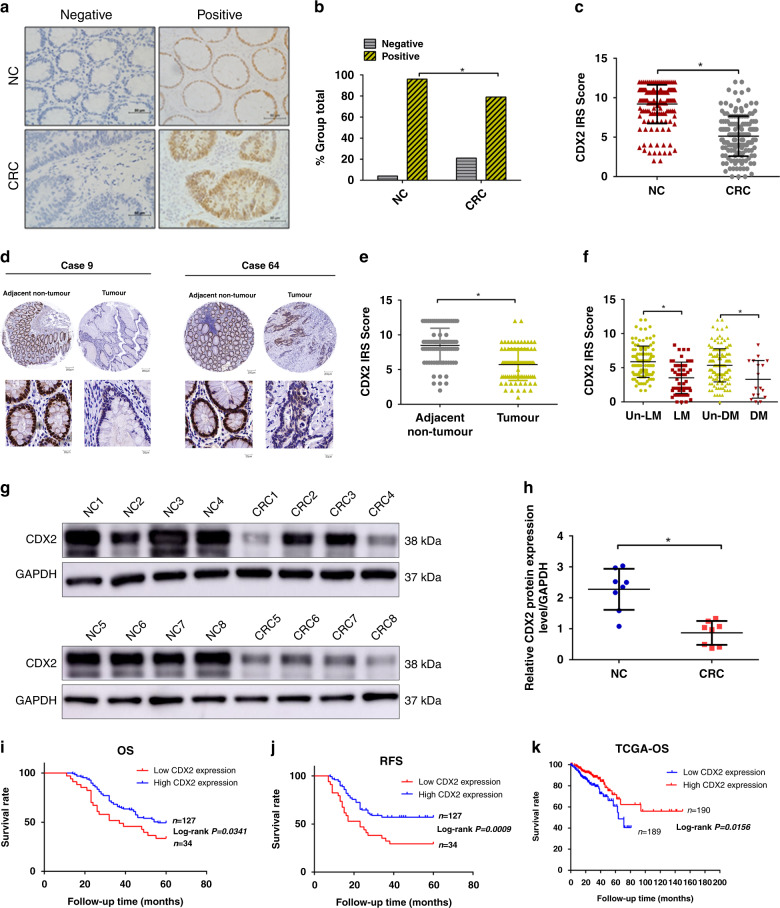
The expression of CDX2 in colorectal cancer (CRC) and normal tissue samples. **a** CDX2 expression in 161 CRC tissue samples and paired NC tissue samples by immunohistochemistry (IHC) staining. **b** The positivity of CDX2 staining in 161 CRC tissue samples and paired NC tissue. **c** The immunoreactivity score (IRS) of CDX2 staining in 161 CRC tissue samples and paired NC tissue. **d** CDX2 expression in tissue microarrays, including 90 pairs of CRC samples and adjacent NC tissues. **e** The IRS of CDX2 staining in tissue microarrays. **f** The IRS of CDX2 staining in CRC tissues with lymphatic or distant metastasis versus CRC tissues without metastasis. LM lymphatic metastasis, DM distant metastasis. **g** Western blot bands for CDX2 in normal and CRC tissue samples. **h** Quantitative analysis of CDX2 expression in normal and CRC tissue samples. **i**, **j** Kaplan–Meier representation of the overall survival (**h**) and recurrence-free survival (RFS) (**i**) of the two groups of patients with high (*n* = 127, blue line) or low (*n* = 34, red line) CDX2 expression in CRC tissues. **k** Data in the TCGA database showed the overall survival of the two groups of patients with high (*n* = 190, red line) or low (*n* = 189, blue line) CDX2 expression in colon cancer tissues. All data are the mean ± SD of three independent experiments. *P < 0.05.

To determine the role of CDX2 in colorectal tumorigenesis, an IHC assay was first performed using 161 pairs of CRC versus adjacent NC tissues. CDX2 staining was observed in the nuclei of positive cells (Fig. 1a). The positive CDX2 expression rates were 95.7% (154/161) in NC tissue samples and 78.9% (127/161) in CRC tissue samples (Fig. 1b). The immunoreactivity score (IRS) of CDX2 staining was reduced in the CRC tissue samples relative to that in the NC tissue samples (Fig. 1c). The expression level of CDX2 was further examined in tissue microarrays, including 90 pairs of CRC samples (Fig. 1d). The average expression of CDX2 was significantly lower in CRC tissues than that in adjacent NC tissues (Fig. 1e). Upregulation of CDX2 was confirmed in 8 paired CRC samples using western blotting (Fig. 1g, h). Intriguingly, the CRC samples with lymphatic or distant metastasis exhibited lower CDX2 expression than those without metastasis (Fig. 1f). Furthermore, the association between CDX2 expression and clinicopathological characteristics in CRC tissue samples was analysed (Supplementary Table 3). CDX2 expression was negatively associated with lymphatic and distant metastasis and TNM staging. Kaplan–Meier analysis showed that patients with low levels of CDX2 expression in tumour had shorter overall survival (OS) and recurrence-free survival (RFS) than those with high levels (Fig. 1i, j). Multivariate analyses validated CDX2 as an independent predictor of OS but not RFS (Supplementary Tables 4 and 5). Analyses from TCGA databases also supported an inverse correlation between CDX2 and OS in the patients with colon cancer (Fig. 1k). In summary, these results indicate that a low level of CDX2 correlates with progression and poor prognosis in human CRC.

### CDX2 inhibits the invasion and metastasis of CRC in vitro and in vivo

Next, western blotting assay was performed to investigate differences in the expression levels of CDX2 in five colon cancer cell lines: RKO, Caco-2, HT-29, SW480 and Lovo (Supplementary Fig. 1a, b). To explore the impact of CDX2 in colorectal tumorigenesis, a series of in vitro and in vivo experiments were conducted in colon cancer cells with gain- and loss of function of CDX2. Depletion of CDX2 in HT-29 and SW480 cells or enhancing CDX2 expression in Lovo and Caco-2 cells were confirmed by western blotting analysis (Supplementary Fig. 1c–f).

**Fig. 2:**
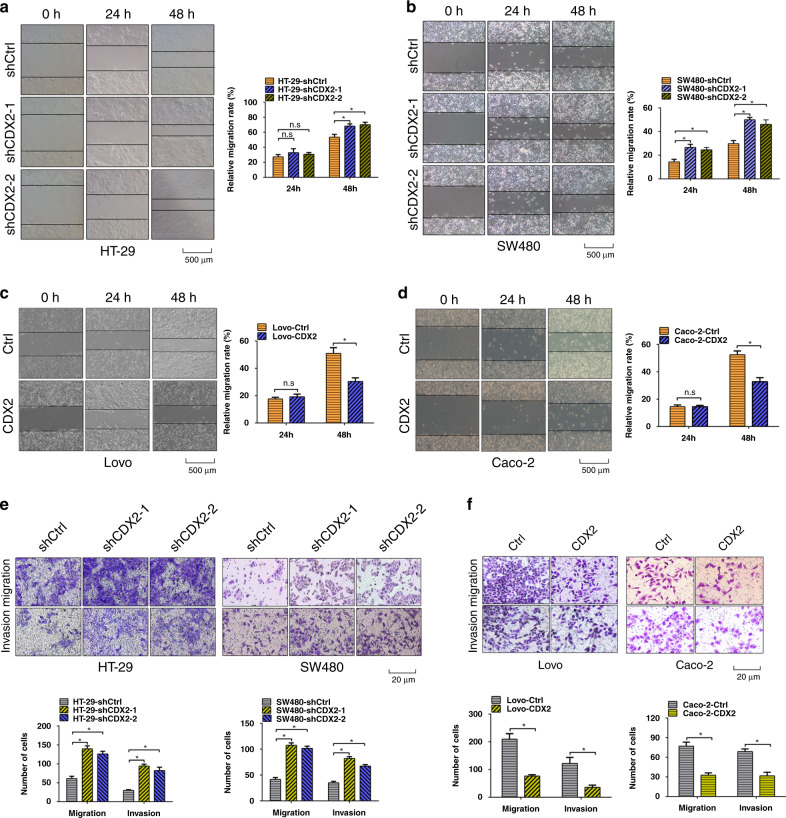
CDX2 inhibits the invasive and migratory abilities of colon cancer cells in vitro. **a**, **b** Wound-healing assay in CDX2-knockdown HT-29 (**a**) and SW480 (**b**) cells. **c**, **d** Wound-healing assay in CDX2-overexpressing Lovo (**c**) and Caco-2 (**d**) cells. **e**, **f** Transwell assays in CDX2-knockdown (**e**) or CDX2-overexpressing (**f**) colon cancer cells. All data are presented as the mean ± SD from three independent experiments. *P < 0.05.

The wound-healing assay indicated that depletion of CDX2 in HT-29 and SW480 cells increased the migratory rate (Fig. 2a, b); however, enhancing CDX2 expression in Lovo and Caco-2 had the opposite effect (Fig. 2c, d). Likewise, transwell assays revealed that HT-29-shCDX2 and SW480-shCDX2 group displayed more invasive and migrating cells than the control group (Fig. 2e), whereas Lovo-CDX2 and Caco-2-CDX2 group had the opposite alteration (Fig. 2f). These findings demonstrated that CDX2 inhibits colon cancer cell invasion and migration in vitro.

Liver metastasis occurs synchronously or metachronously in approximately 50% of patients with CRC, which directly leads to a poor prognosis.^36^ In this study, colon cancer liver metastasis models were conducted to evaluate the impact of CDX2 depletion in tumour metastasis. Depletion of CDX2 markedly elevated the number of metastatic nodules in the liver (Supplementary Fig. 2a–c). In addition, the weight and survival time of the nude mice in CDX2-depletion group were lower and shorter than those in the control group, respectively (Supplementary Fig. 2d, e). These results indicated that knockdown of CDX2 enhances the metastatic potential of colon cancer cells.

### CDX2 suppresses EMT in CRC

**Fig. 3:**
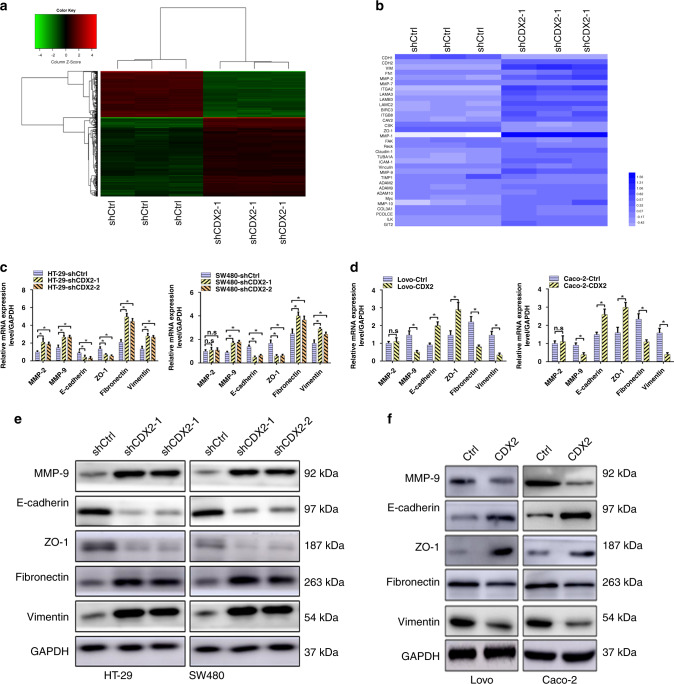
CDX2 inhibits epithelial–mesenchymal transition (EMT) in colorectal cancer (CRC). **a** Hierarchical clustering of genes that were significantly and differentially expressed in SW480-shCDX2 and the control cells. Data were log2 normalised. **b** Visualisation of differentially expressed genes for cellular conjunctions, focal adhesion, cytoskeleton and extracellular matrix of Gene Ontology Enrichment analysis in SW480-shCDX2 and the control cells. **c**, **d** Real-time PCR results of EMT-related genes in CDX2-knockdown (**c**) and CDX2-overexpressing (**d**) cells. **e**, **f** Western blotting bands for EMT-related proteins in CDX2-knockdown (**e**) and CDX2-overexpressing (**f**) cells. All data are presented as the mean ± SD from three independent experiments. *P < 0.05.

To explore the molecular mechanism of CDX2 in CRC metastasis, microarray analysis of SW480-shCDX2 and the control cells was applied (Fig. 3a). Gene Ontology Enrichment analysis identified 39 EMT-related genes (Fig. 3b), which were involved in the cellular conjunctions, focal adhesion, cytoskeleton and the extracellular matrix.^37^

Of the above 39 genes, six classic EMT-related genes, including E-cadherin, vimentin, fibronectin, ZO-1 and MMP-2/9, were further chosen for validation by using real-time PCR and western blotting. The results showed an increased fibronectin, vimentin and MMP-9 levels, and a decreased ZO-1 and E-cadherin levels in CDX2-knockdown cells (Fig. 3c, e and Supplementary Fig. 3a). Conversely, CDX2-overexpressing cells had the reverse change (Fig. 3d, f and Supplementary Fig. 3b). IF (Supplementary Fig. 4a–d) and ICC (Supplementary Fig. 4e, f) assays observed a decline in E-cadherin staining and an increase in vimentin staining in HT-29 and SW480 with CDX2 depletion. Likewise, IHC assay revealed that liver metastatic nodules from HT-29-shCDX2 and SW480-shCDX groups had a much stronger vimentin staining and a weaker E-cadherin staining than those from the control groups (Supplementary Fig. 4g, h).

Furthermore, the levels of several EMT-related transcription factors, including Snail, Slug, Twist and ZEB1/2, were detected. Depletion of CDX2 resulted in a high elevation of Snail level and a moderate elevation of Slug and ZEB1 levels (Supplementary Figs. 3c and 5a). Conversely, enhancing CDX2 expression in Lovo and Caco-2 cells had the opposite effects (Supplementary Figs. 3d and 5b). There was no marked alteration in ZEB2 and Twist expression. We next investigated whether Snail participates in CDX2-inhibited EMT and invasion in CRC. The depletion of Snail neutralised the promoting effect of CDX2 knockdown on invasion and migration of CRC (Supplementary Fig. 5c). Moreover, the depletion of Snail promoted E-cadherin and ZO-1 expression, as well as reduced vimentin and MMP-9 expression (Supplementary Figs. 3e, f and 5d). Snail has been proved to induce MMP-9 expression in tumour invasion.^38^ Taken together, these findings demonstrated that CDX2 antagonises EMT in CRC by regulating Snail, Slug or ZEB1 expression.

### CDX2 inhibits Snail expression through suppressing PI3K/Akt/GSK-3β activity

**Fig. 4:**
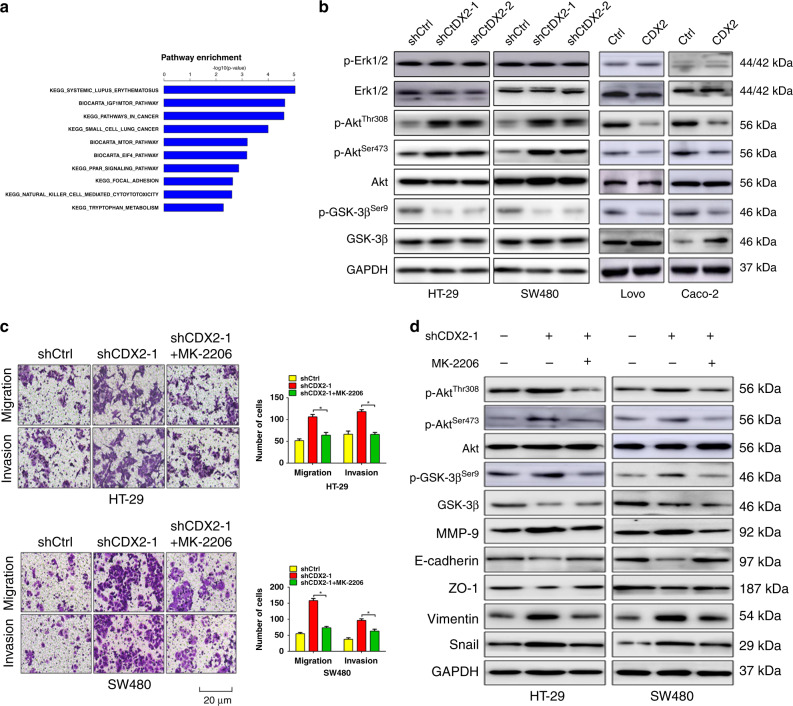
CDX2 inhibits Snail expression through suppressing PI3K/Akt/GSK-3β activity. **a** Pathway analysis of genes that were significantly and differentially expressed in SW480-shCDX2 and control cells using KEGG database. **b** Western blotting bands for AKT/p-AKT (Thr308/Ser473), GSK-3β/p-GSK-3β (Ser9) and Erk1/2/p-Erk1/2 (Thr202/Tyr204) in CDX2-knockdown and -overexpressing cells. **c** Transwell assays in CDX2-knockdown cells treated with MK-2206. **d** Western blotting bands for PI3K/Akt signalling- and EMT-related proteins and in CDX2-knockdown cells treated with MK-2206. All data are presented as the mean ± SD from three independent experiments. *P < 0.05.

PI3K/Akt and MAPK/Erk pathways play a crucial role in colorectal carcinogenesis.^39,40^ Previous study indicated that CDX2 level negatively correlated with the activity of PI3K/Akt pathway.^41,42^ Moreover, microarray analysis via KEGG software indicated that PI3K/Akt/MTOR had the marked change in all relevant signalling pathways (Fig. 4a). We first examined the effect of CDX2 on the activity of PI3K/Akt and MAPK/Erk pathways. The results showed that modulation of CDX2 expression had little effect on Erk phosphorylation (Fig. 4b and Supplementary Fig. 6a, b). However, depletion of CDX2 induced phosphorylation of Akt (Thr308/Ser473) and GSK-3β (Ser9), whereas enhancing CDX2 expression had the opposite alteration (Fig. 4b and Supplementary Fig. 6a, b). Intriguingly, we found that knockdown or ectopic expression of CDX2 reduced or elevated the expression of GSK-3β, respectively. Our previous study indicated that CDX2 can regulate GSK-3β transcription by directly binding to the GSK-3β promoter.^34^

GSK-3β was previously reported to mediate Snail stabilisation.^43^ We thus attempt to evaluate whether the activity of PI3K/Akt pathway affected CDX2-regulated Snail expression and invasion in CRC. Blockade of PI3K/Akt activity by MK-2206 diminished the effect of CDX2 knockdown on invasion (Fig. 4c) and Snail expression (Fig. 4d and Supplementary Fig. 6c, d). Moreover, treatment with MK-2206 promoted E-cadherin and ZO-1, as well as downregulated vimentin and MMP-9. Altogether, these results indicate that CDX2 antagonises EMT in CRC by suppressing PI3K/Akt/GSK-3β activity and Snail expression.

### CDX2 destabilises β-catenin in CRC through the PI3K/Akt/GSK-3β pathway

**Fig. 5:**
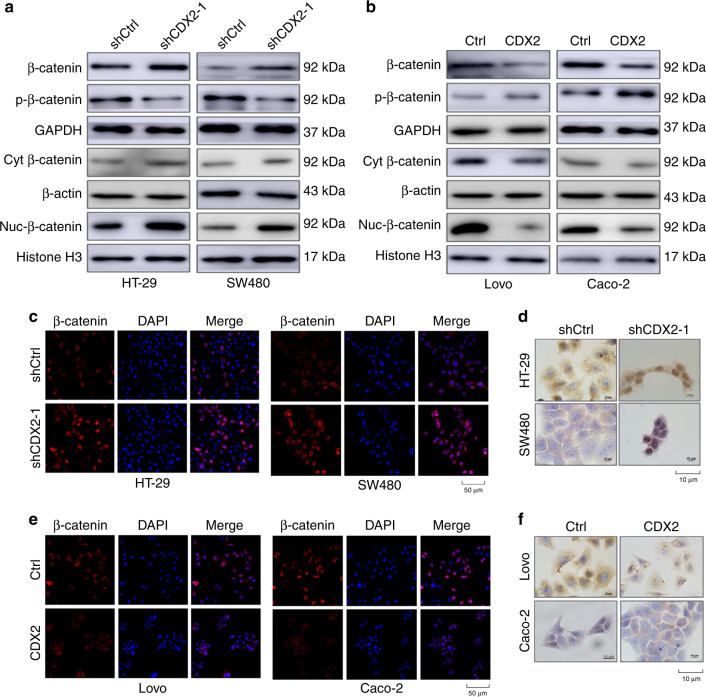
CDX2 inhibits the stabilisation and nuclear translocation of β-catenin in colorectal cancer (CRC). **a**, **b** Western blotting bands for nuclear β-catenin and total/phospho-β-catenin protein in CDX2-knockdown (**a**) and CDX2-overexpressing (**b**) cells. **c**, **e** IF staining of β-catenin in CDX2-knockdown (**c**) and -overexpressing (**e**) cells. Scale bar, 50 μm. **d**, **f** ICC staining of β-catenin in CDX2-knockdown (**d**) and -overexpressing (**f**) cells. Scale bar, 10 μm. All data are presented as the mean ± SD from three independent experiments.

An elevated nuclear β-catenin can induce the expression of its target genes, including Slug, ZEB1 and MMP-7, thereby promoting EMT and tumour cell metastasis.^22,44^ Our study observed that knockdown of CDX2 elevated total β-catenin and its nuclear translocation (Fig. 5a and Supplementary Fig. 7a), which was further confirmed by IF and ICC assays (Fig. 5c, d), while ectopic expression of CDX2 had the reserved change (Fig. 5b, e, f and Supplementary Fig. 7a). GSK-3β-induced β-catenin phosphorylation and degradation is the primary mechanism of regulating β-catenin levels.^45^ Active Akt can phosphorylate GSK-3β at Ser9 and impede its activity.^20^ Thus, we hypothesised that CDX2 regulates EMT through its effects on GSK-3β phosphorylation and downstream effects on β-catenin. First, we detected the effect of modulating CDX2 expression on stabilisation of β-catenin. Knockdown of CDX2 diminished the phosphorylation of β-catenin (Ser33/37/Thr41), while ectopic expression of CDX2 enhanced phosphorylated β-catenin (Fig. 5a, b and Supplementary Fig. 7a). Moreover, the amount of β-catenin accumulated sharply in HT-29 and SW480 cells with the proteasome inhibitor MG132 treatment (Supplementary Fig. 8a). The cycloheximide (CHX) pulse-chase assay showed that depletion of CDX2 lengthened the half-life of β-catenin protein, and enhanced CDX2 expression-accelerated β-catenin degradation (Supplementary Fig. 8b–e). To further demonstrate that CDX2 exerts its function through GSK-3β, a GSK-3β inhibitor CHIR-98014 was used. CHIR-98014 rescued the effect of CDX2 overexpression on β-catenin (Supplementary Figs. 7b and 9a). Moreover, inhibition of the PI3K/Akt pathway by MK-2206 confirmed that the PI3K/AKT/GSK-3β pathway is involved in CDX2-regulated β-catenin levels (Supplementary Figs. 7c and 9b).

In addition, the depletion of β-catenin diminished the effect of CDX2 knockdown on invasion and migration of CRC (Supplementary Fig. 9c). As previously reported, Slug and ZEB1 might be two of the target genes of β-catenin in CRC.^22^ The depletion of β-catenin reduced Slug, ZEB1 and vimentin, as well as elevated E-cadherin and ZO-1 (Supplementary Fig. 7d and 9d). There was no significant alteration in Snail expression. Altogether, our study indicated that CDX2 destabilises β-catenin through the PI3K/Akt/GSK-3β pathway.

### CDX2 attenuates PI3K/Akt activity in CRC by regulating PTEN expression

Previous study has identified the co-expression levels of PTEN and CDX2 in gastric cancer.^41^ PTEN has attracted our attention because of its role in the dephosphorylation of Akt. Thus, we speculate that CDX2 inhibits Akt phosphorylation via regulating PTEN expression. The results showed that depletion of CDX2 inhibited the expression of PTEN at both mRNA and protein levels, whereas enhancing CDX2 expression had the reverse change (Supplementary Figs. 10a, b and 11a, b). To further determine the role of PTEN in CDX2-inhibited Akt phosphorylation and invasion of colon cancer cells, we ectopically expressed or knocked down PTEN in colon cancer cells with stable CDX2 knockdown or overexpression. Ectopic expression of PTEN significantly diminished tumour cell invasion as well as Akt phosphorylation and EMT marker proteins induced by CDX2 knockdown (Supplementary Figs. 10c, e and 11c, d); conversely, knockdown of PTEN recovered the tumour-suppressive effect of CDX2 overexpression (Supplementary Figs. 10d, f and 11e, f). Taken together, these data indicated that CDX2 suppresses PI3K/Akt activity in CRC by regulating PTEN expression.

### PTEN was identified as a downstream target of CDX2

**Fig. 6:**
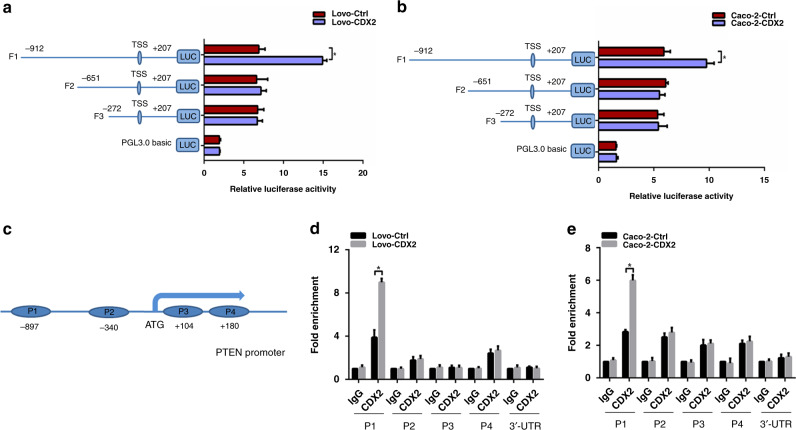
CDX2 transactivates the expression of PTEN by binding to the PTEN promoter in colon cancer cells. **a**, **b** The activities of the PTEN full promoter reporter construct and the truncated ones in CDX2-overexpression Lovo (**a**) and Caco-2 (**b**) cells using the dual-luciferase assay. **c** Schematic representation of the CDX2 putative binding sites (P1–P4) in PTEN promoter region. **d**, **e** Enrichment level of the CDX2- binding putative site in the PTEN promoter region in Lovo (**d**) and Caco-2 (**e**) cells determined by the qChIP assay. All data are presented as the mean ± SD from three independent experiments. *P < 0.05.

We further assessed whether CDX2 suppresses the PI3K/Akt pathway via transcriptional activation of PTEN. First of all, the full-length PTEN promoter (from –912 bp to +207 bp) reporter construct and the other three truncated ones were constructed and transfected into Lovo-CDX2 and Caco-2-CDX2 and the control cells, respectively. The luciferase activity of the PTEN promoter was detected by dual-luciferase reporter assay. Ectopic expression of CDX2 resulted in an elevated luciferase activity of the full-length fragments, but did not affect the luciferase activity of the other truncated fragments (Fig. 6a, b), suggesting that CDX2 could transactivate PTEN expression by binding to the −912 bp to −651 bp of the PTEN promoter. Next, we attempted to confirm whether CDX2 protein binds to the special site of the PTEN promoter in vivo by using qChIP assay. Four pairs of primers were designed to amplify the four P1–P4 fragments of the −912-bp to +207-bp PTEN promoter region (Fig. 6c). The results showed that ectopic expression of CDX2 enhanced the binding of CDX2 to P1 promoter fragment but not the P2–4 promoter fragments (Fig. 6d, e). All these results indicated that CDX2 could bind to the P1 fragment of the PTEN promoter and transcriptionally activate PTEN in colon cancer cells.

### Correlations among CDX2, PTEN, Snail, E-cadherin and vimentin expression levels in CRC tissues

The clinical relevance of CDX2, PTEN and EMT marker proteins in 46 randomly selected CRC tissues was explored (Supplementary Fig. 12a). We found that CDX2 expression was positively associated with PTEN and E-cadherin, and was negatively associated with Snail and vimentin in CRC specimens (Supplementary Fig. 12b–e). These results further support the notion that CDX2 acts as a negative regulator of EMT in colon cancer cells.

## Discussion

Emerging evidence supports a crucial role for CDX2 as a tumour suppressor in colorectal tumorigenesis. However, the exact function of CDX2 during the induction of EMT in CRC remains to be elucidated. Our results gain new insight into the role of CDX2 in EMT. The present study firstly revealed that CDX2 expression was lower in CRC than NC tissue samples. Moreover, the expression of CDX2 is inversely correlated with lymphatic and distant metastasis and TNM staging. In stage IV unresectable CRC, lack of CDX2 expression predicted poor survival.^46^ Moreover, lack of CDX2 expression was proposed as a poor prognostic and predictive biomarker for the response to chemotherapy in stages II and III CRC.^47^ Consistently, our data indicated that reduced CDX2 correlated with worse OS and RFS of patients with CRC. Moreover, CDX2 was validated as an independent predictor of OS but not RFS. In summary, CDX2 is likely to be an important biomarker for guiding evaluation of tumour progression and prognosis.

Distant metastasis and relapse directly lead to a poor prognosis of CRC patients.^48^ In this study, the results obtained in liver metastasis models showed that knockdown of CDX2 promoted CRC liver metastasis in vivo. Subsequently, we demonstrated that CDX2 inhibited colon cancer cell invasion and migration in vitro. EMT has been linked to the mobility and dissemination of CRC by conferring increased invasiveness and metastatic potential to cells.^49,50^ By microarray analysis, real-time PCR and western blotting validation, we observed that colon cancer cells with CDX2 knockdown expressed high levels of fibronectin, vimentin and MMP-9, and low levels of ZO-1 and E-cadherin. Enhancing CDX2 expression had the reversed EMT programme. These data support that deficiency of CDX2 might be involved in metastasis of CRC through promoting EMT.

Next, the molecular mechanism of EMT phenotypic changes was explored. There were a variety of signalling pathways responsible in regulating EMT, including Wnt/β-catenin, tumour growth factor, Notch and PI3K/Akt pathways.^13–15^ Our study revealed that knockdown of CDX2 induced the phosphorylation of Akt and GSK-3β and promoted the expression of Snail, while ectopic expression of CDX2 had the opposite effect. It has been reported that active Akt impedes GSK-3β phosphorylation, which results in the stabilisation of Snail.^43^ In our study, blockade of PI3K/Akt pathway by MK-2206 in CDX2-depleted cells increased phosphorylation of GSK-3β, which was accompanied by Snail suppression. In summary, we concluded that CDX2 antagonises EMT in CRC by suppressing Snail expression through PI3K/Akt/GSK-3β pathway. Consistently, depletion of CDX2 in lung cancer promoted cell invasion and metastasis by increasing Snail expression.^51^ Intriguingly, Gross et al. reported that Snail could repress CDX2 transcription in colon cancer cells.^52^ Our previous study also indicated that Snail is involved in the HIF-α-induced downregulation of CDX2,^53^ implying that a positive feedback mechanism might exist between CDX2 and Snail; however, the exact mechanism remains to be further elucidated.

The PI3K/Akt pathway impedes the activity of GSK-3β, leading to the stabilisation and nuclear translocation of β-catenin to promote cell proliferation, differentiation and EMT.^20^ Here, we reported that CDX2 knockdown promoted the stabilisation and nuclear translocation of β-catenin in CRC, while ectopic expression of CDX2 had the reserved alterations. Nuclear accumulation of β-catenin interacts with LEF/Tcf transcriptional factors, which induces the transcription of EMT-related genes.^54^ Expectedly, the levels of Slug and ZEB1, two target genes of β-catenin, were increased in response to CDX2 knockdown, and this effect can be reversed by the depletion of β-catenin. Previous study indicated that direct phosphorylation of β-catenin by AKT also promotes its nuclear translocation and increases the transcriptional activity.^55^ Snail, Slug and ZEB1 acts as a strong inducer of EMT by repressing E-cadherin transcription.^11,56^ In our study, an elevation of Snail, Slug and ZEB1 followed by CDX2 knockdown might retard E-cadherin/β-catenin complex and induce β-catenin release from the membrane, which was confirmed by the results of IF assay showing a decreased β-catenin in the membrane of CDX2-depleted cells. Deficiency of membrane localisation of β-catenin causes the disassociation of cell–cell contracts and enhanced the metastatic potential of CDX2-depleted cells. Collectively, our study demonstrated that CDX2 inhibits EMT and metastasis of CRC by regulation of Snail expression and β-catenin stabilisation through PI3K/Akt/GSK-3β signalling. During gastrin-induced migration, Fas-induced EMT and endothelin-1-mediated EMT and tumour invasion, the inactivation of GSK-3β by PI3k/Akt signalling promotes Snail expression and β-catenin stabilisation.^57,58^

PTEN acts as negative regulator of PI3K-mediated AKT activation in cell homoeostasis.^59^ Inactivation of PTEN has been reported to participate in EMT acquisition during the process of tumour metastasis.^60^ Emerging evidence indicated that PTEN can be regulated at the transcriptional level as well as by numerous post-transcriptional modifications.^59^ We observed that CDX2 regulated PTEN expression at both mRNA and protein levels. Moreover, ectopic expression of PTEN attenuated tumour cell invasion as well as Akt and GSK-3β phosphorylation and EMT marker proteins enhanced by CDX2 knockdown, while knockdown of PTEN antagonised the tumour-suppressive effect of CDX2 overexpression. The dual-luciferase reporter assay confirmed that CDX2 could promote PTEN transcription by binding to the −912-bp to −651-bp region of the PTEN promoter. Mechanistically, CDX2 specifically and directly binds to the P1 fragment of the PTEN promoter detected by qChIP. This is the first study to identify that CDX2 could directly regulate PTEN transcription.

This study indicated that depletion of PTEN elevated the expression of both total and nuclear β-catenin in CDX2-overexpressed cells. The direct target of PTEN by EBV-miR-BART7-3p exhibits a similar alteration in nasopharyngeal carcinoma.^14^ However, Elumalai et al. reported that the level of total β-catenin was reduced in PTEN-inactivated lung cancer cells, despite the accumulation of β-catenin in cell nucleus.^60^ These studies unanimously concluded that PTEN inactivation in tumour induces the nuclear accumulation of β-catenin, although the total β-catenin level might exhibit the inconsistent alteration. This might be due to loss of β-catenin substrate protein E-cadherin or proteasomal degradation of β-catenin substrate by AKT-mediated phosphorylation. Intriguingly, inhibition of PTEN by Hes-1 resulted in a decrease in the level of membrane and cytoplasmic β-catenin, but not nuclear accumulation.^61^

In this study, by conducting in vitro and in vivo assays and using a group of CRC patients, we first showed that CDX2 is a major inhibitor of the invasion-prone phenotype and EMT in colon cancer cells. Second, we demonstrated that CDX2 could directly transactivate PTEN expression and thereby suppress PI3K/Akt/GSK-3β signalling, which results in decreasing Snail expression and destabilising β-catenin. Decreased nuclear β-catenin suppresses Slug and ZEB1 transcription, and the reduction of Snail, Slug and ZEB1 induces E-cadherin transcription, which consequently enhances cell–cell interaction and retards cell migration and invasion. Finally, in clinical CRC samples, we observed that CDX2 was positively correlated with PTEN and E-cadherin expression, and was negatively correlated with Snail and vimentin expression. In summary, these findings reveal that CDX2 exerted an inhibitory impact on EMT and metastasis in CRC. The pivotal signalling pathway involved in this process was identified, as were suitable candidates for therapeutic targets in CRC patients.

## Supplementary information

Supplementary file

## Data Availability

The datasets generated and/or analysed during the current study are not publicly available but are available from the corresponding author on reasonable request.
