# Supplementary material for: CDX2 inhibits epithelial–mesenchymal transition in colorectal cancer by modulation of Snail expression and β-catenin stabilisation via transactivation of PTEN expression
Source: Br J Cancer. 2020 Nov 26;124(1):270–80. doi: 10.1038/s41416-020-01148-1 (PMC7782852; doi:10.1038/s41416-020-01148-1)
Supplement: Supplementary file 1 — Supplementary file [file 41416_2020_1148_MOESM1_ESM.docx]

**Supplementary Fig. 1 The effects of up-regulation and down-regulation of CDX2 expression in colon cancer cells**


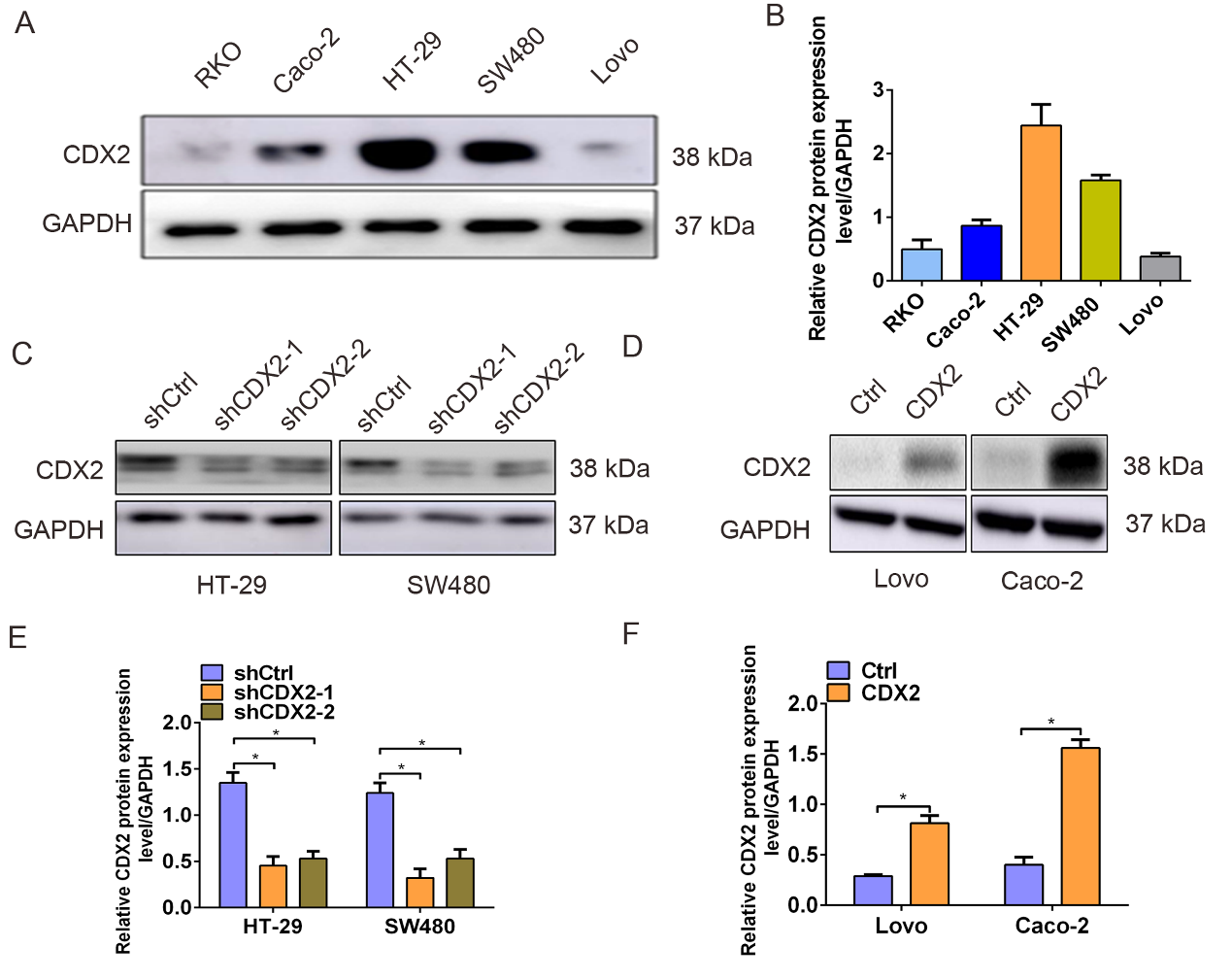


**Supplementary Fig. 1 The effects of up-regulation and down-regulation of CDX2 expression in colon cancer cells.** **a** Western blotting bands for CDX2 in five colon cancer cell lines: RKO, Caco-2, HT-29, SW480 and Lovo. **b** Quantitative analysis of CDX2 expression in four colon cancer cell lines. **c, d** Western blotting bands for CDX2 in CDX2-knockdown (**c**) and CDX2-overexpressing (**d**) cells. **e, f**. Quantitative analysis of CDX2 expression in CDX2-knockdown (**e**) and CDX2-overexpressing (**f**) cells. All data are the mean±SD of three independent experiments. **P*<0.05.

**Supplementary Fig. 2 Knockdown of CDX2 promotes the liver metastasis of colon cancer cells in vivo**


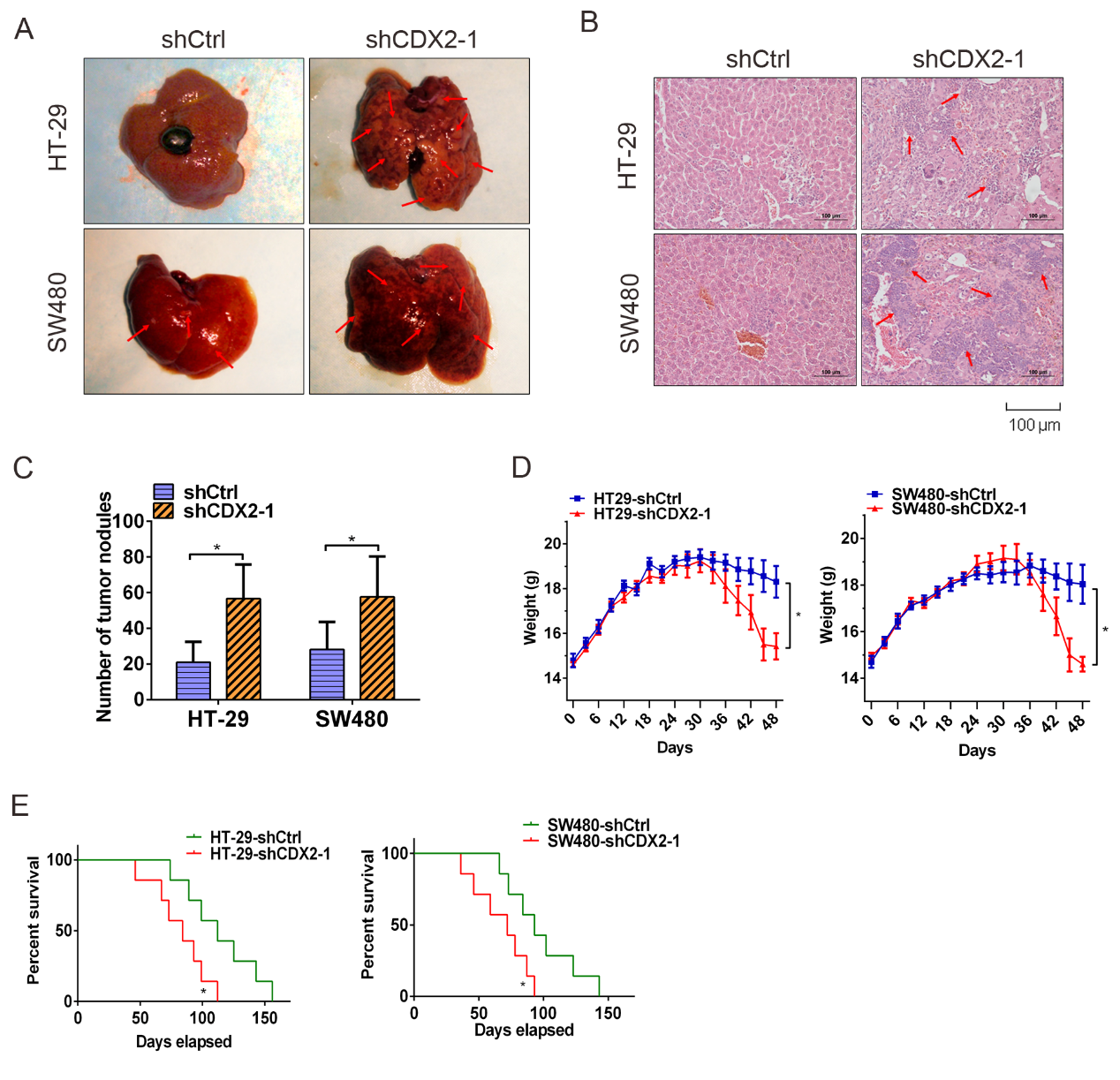


**Supplementary Fig. 2 Knockdown of CDX2 promotes the liver metastasis of colon cancer cells in vivo**. **a** The metastases in liver were shown for CDX2-knockdown group and the control group. Liver metastasis models of colon cancer were generated in BALB/c-nude mice with CDX2-knockdown HT-29 and SW480 cells via intrasplenic injection. **b** H-E staining of tumor nodules (red arrow) in liver metastasis in CDX2-knockdown group and the control group. **c** Average number of tumor nodules in liver metastasis in CDX2-knockdown group and its control group. **d** The weight of nude mice in CDX2-knockdown group and the control group. **e** The survival time of the nude mice in CDX2-knockdown group and the control group. **P* <0.05.

**Supplementary Fig. 3 CDX2 inhibits EMT in CRC by regulating Snail expression**


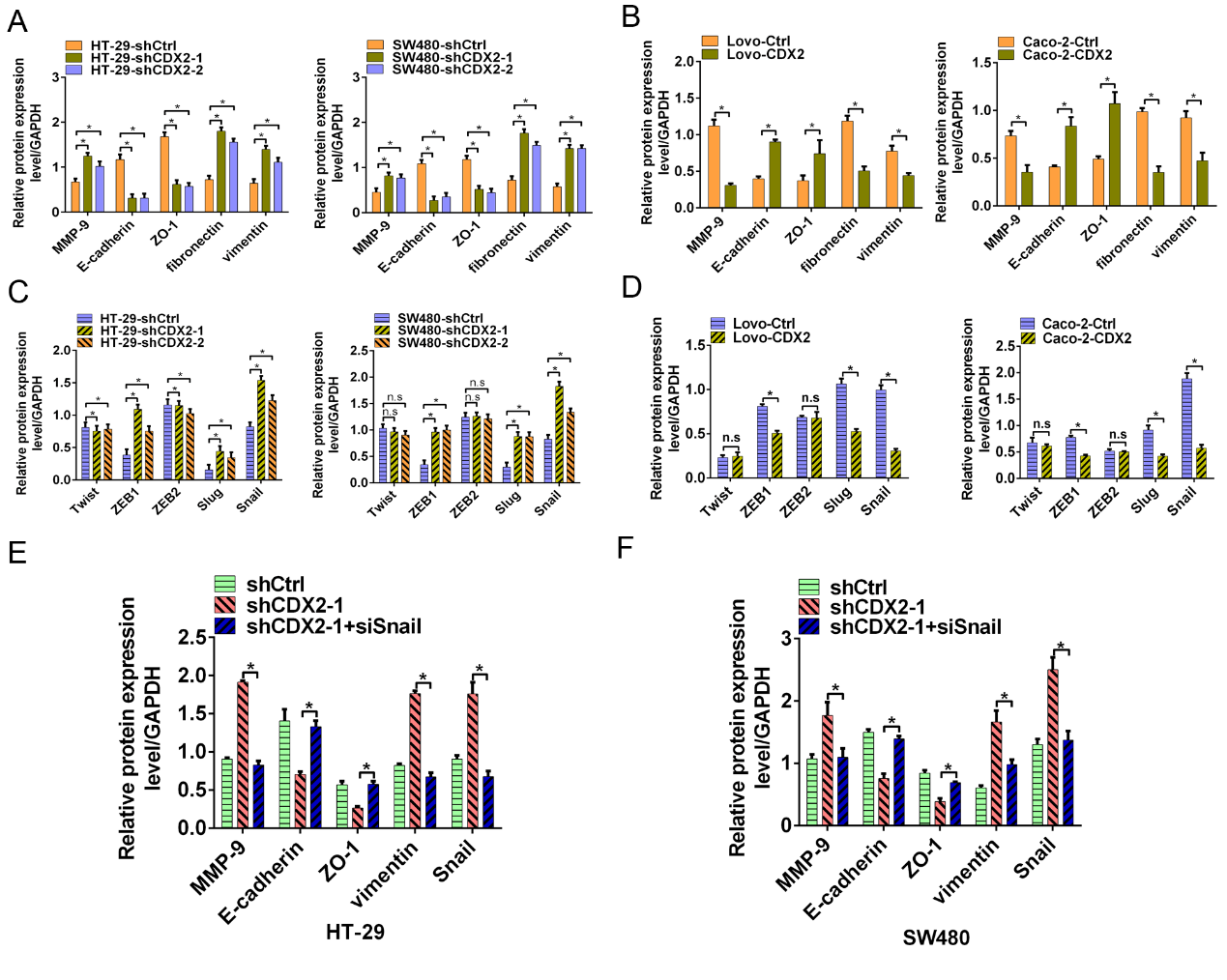


**Supplementary Fig. 3 CDX2 inhibits EMT in CRC by regulating Snail expression. a, b** Quantitative analysis of EMT-related proteins in CDX2-knockdown (**a**) and CDX2-overexpressing (**b**) cells. **c, d** Quantitative analysis of EMT-related transcription factors in CDX2-knockdown (**c**) and CDX2-overexpressing (**d**) cells. **e, f** Quantitative analysis of EMT-related proteins in CDX2-knockdown HT-29 (**e**) and SW480 (**f**) cells transfected with siSnail. All data are presented as the mean±SD from three independent experiments. **P* <0.05.

**Supplementary Fig. 4 CDX2 inhibits EMT in CRC**


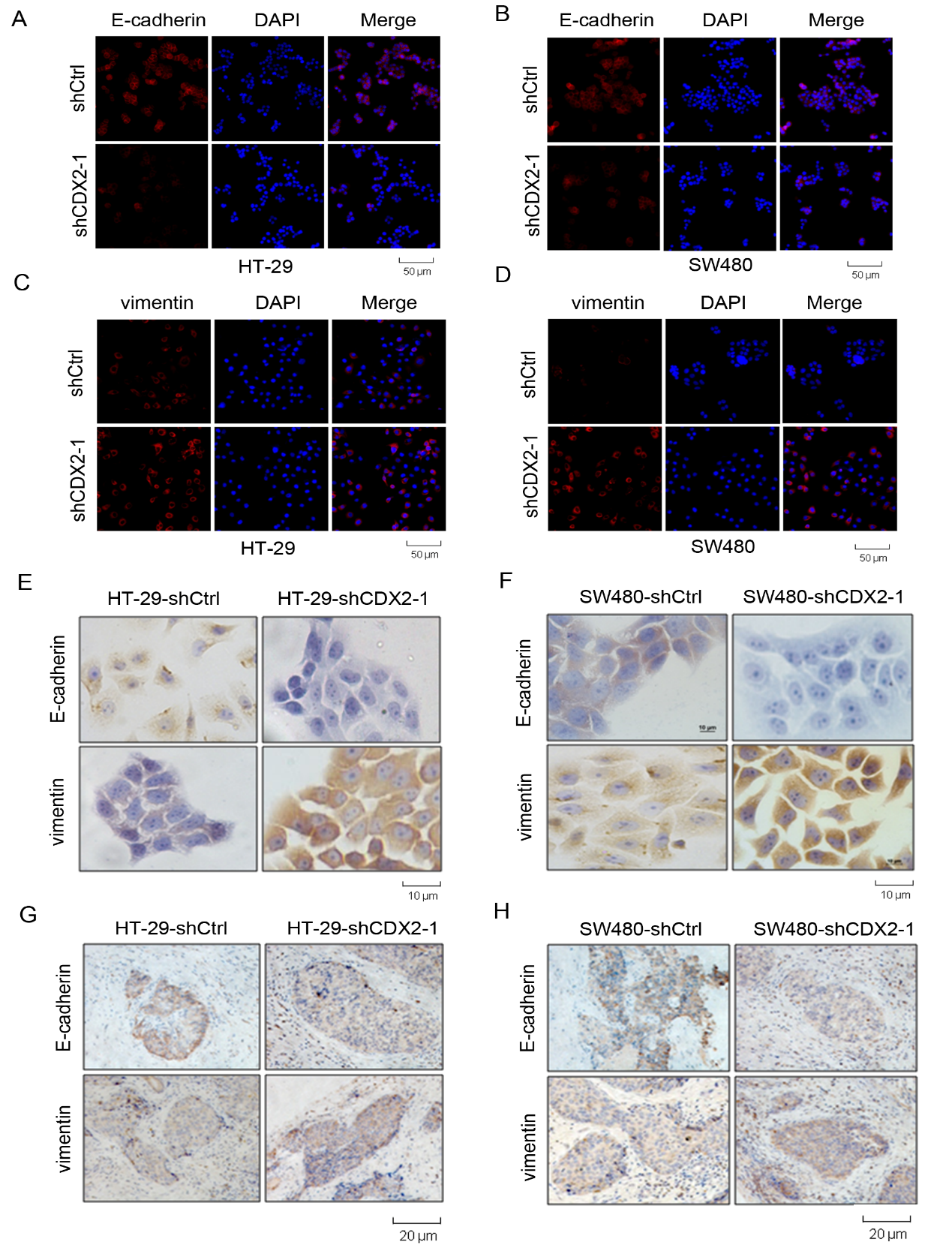


**Supplementary Fig. 4 CDX2 inhibits EMT in CRC**. **a, b** Immunofluorescence (IF) staining of E-cadherin in CDX2-knockdown HT-29 (**a**) and SW480 (**b**) cells. Scale bars, 50 μm. **c, d** IF staining of vimentin in CDX2-knockdown HT-29 (**c**) and SW480 (**d**) cells. Scale bars, 50 μm. **e, f** Immunocytochemistry (ICC) staining of E-cadherin and vimentin in CDX2-knockdown HT-29 (**e**) and SW480 (**f**) cells. Scale bars, 10 μm **g, h** Immunohistochemistry (IHC) staining of E-cadherin and vimentin in liver metastatic tumor tissues formed by CDX2-knockdown HT-29 (**g**) and SW480 (**h**) cells and the control cells. Scale bars, 20 μm. All data are presented as the mean±SD from three independent experiments. **P* <0.05.

**Supplementary Fig. 5 CDX2 suppresses EMT by regulating Snail expression**


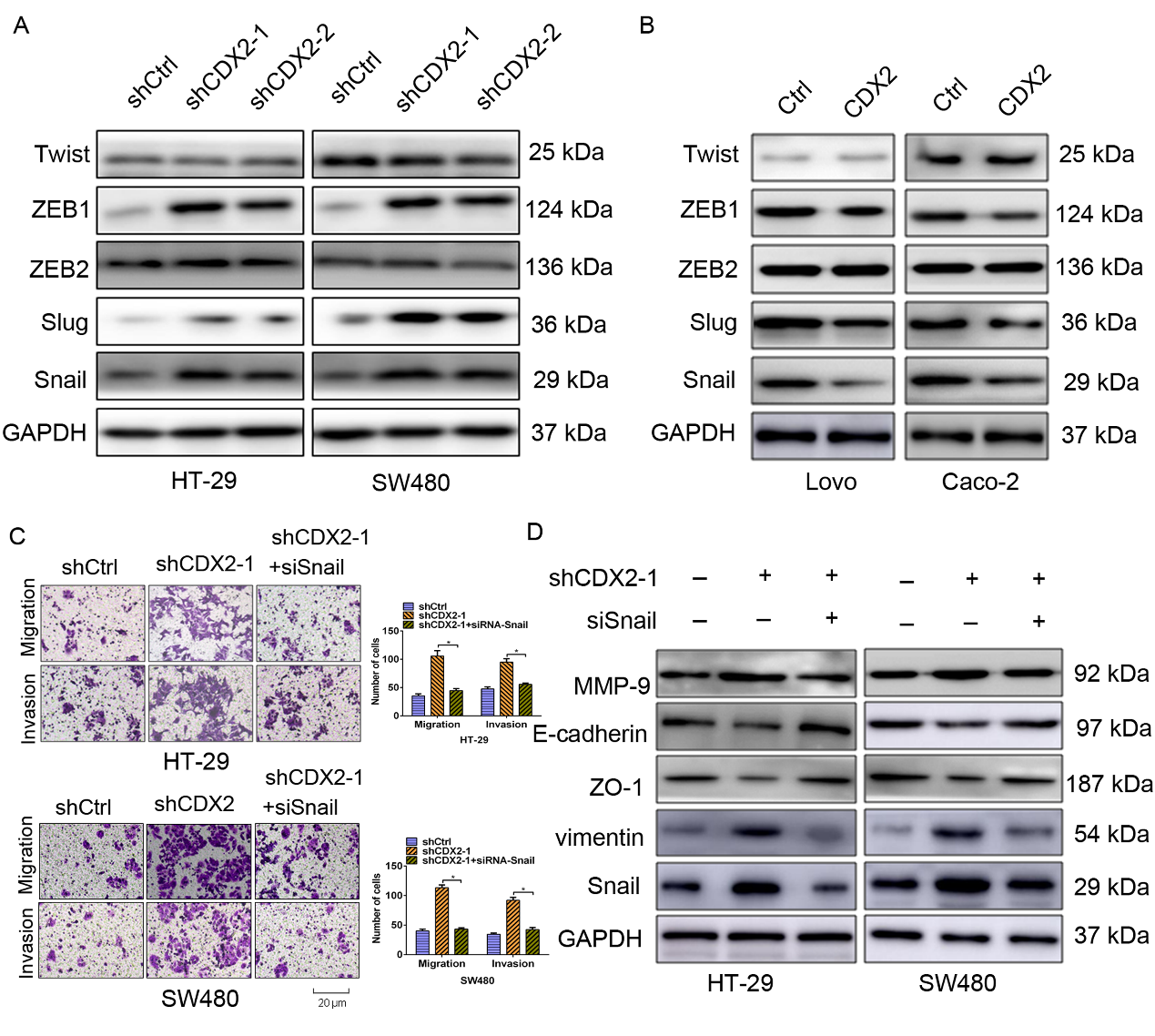


**Supplementary Fig. 5 CDX2 suppresses EMT by regulating Snail expression. a, b** Western blotting bands for EMT-related transcription factors in CDX2-knockdown (**a**) and CDX2-overexpressing (**b**) cells. **c** Transwell assays in CDX2-knockdown HT-29 and SW480 cells transfected with siRNA-Snail. **d** Western blotting bands for EMT-related proteins in CDX2-knockdown cells transfected with siRNA-Snail. All data are presented as the mean±SD from three independent experiments. **P* <0.05.

**Supplementary Fig. 6 CDX2 inhibits Snail expression through suppressing PI3K/Akt/GSK-3β activity**


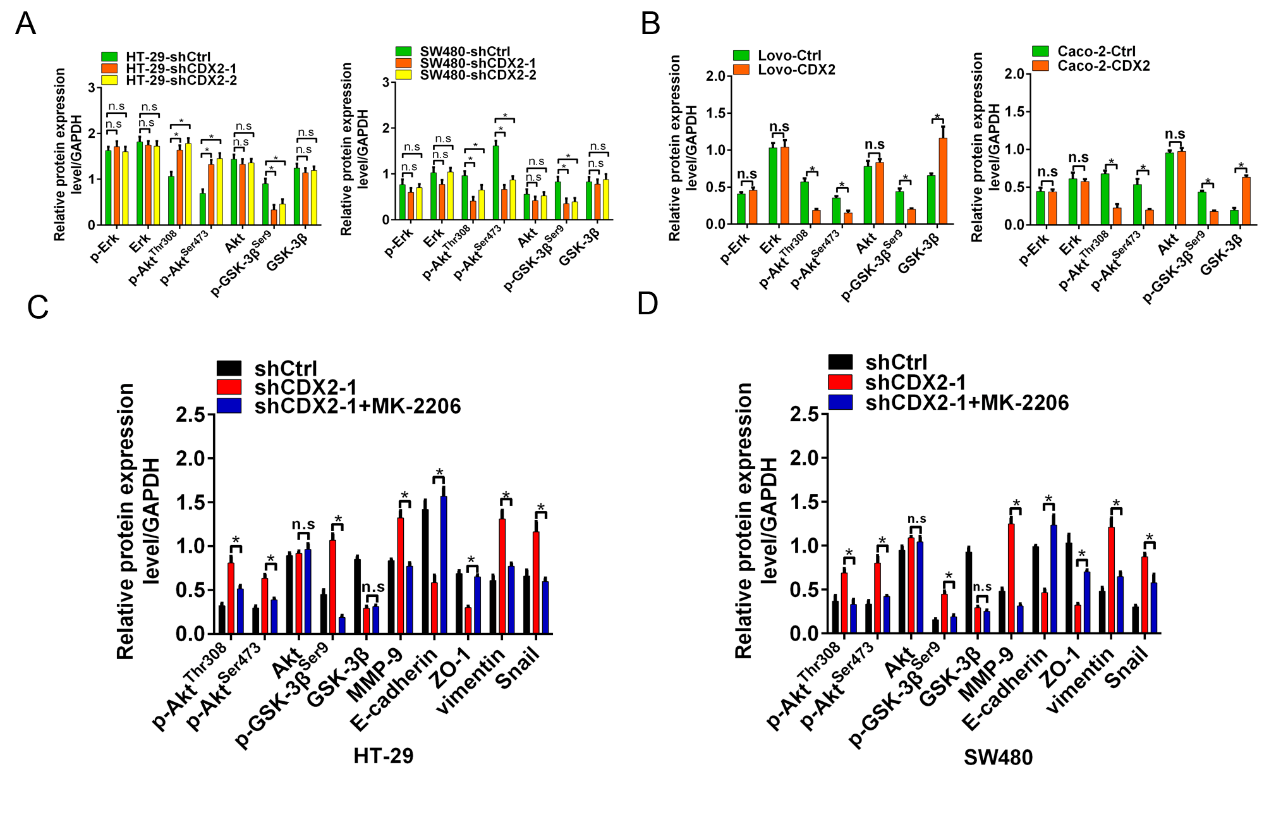
**Supplementary Fig. 6 CDX2 inhibits Snail expression through suppressing PI3K/Akt/GSK-3β activity. a, b** Quantitative analysis of AKT/p-AKT (Thr308/Ser 473), GSK-3β/p-GSK-3β (Ser9) and Erk1/2/p-Erk1/2 (Thr202/Tyr204) in CDX2-knockdown (**a**) and CDX2-overexpressing (**b**) cells. **c, d** Quantitative analysis of for PI3K/Akt signaling-related proteins and EMT-related proteins and in CDX2-knockdown HT-29 (**c**) and SW480 (**d**) cells treated with MK-2206. All data are presented as the mean±SD from three independent experiments. **P* <0.05.

**Supplementary Fig. 7 CDX2 destabilizes β-catenin in CRC through PI3K/Akt/GSK-3β pathway**


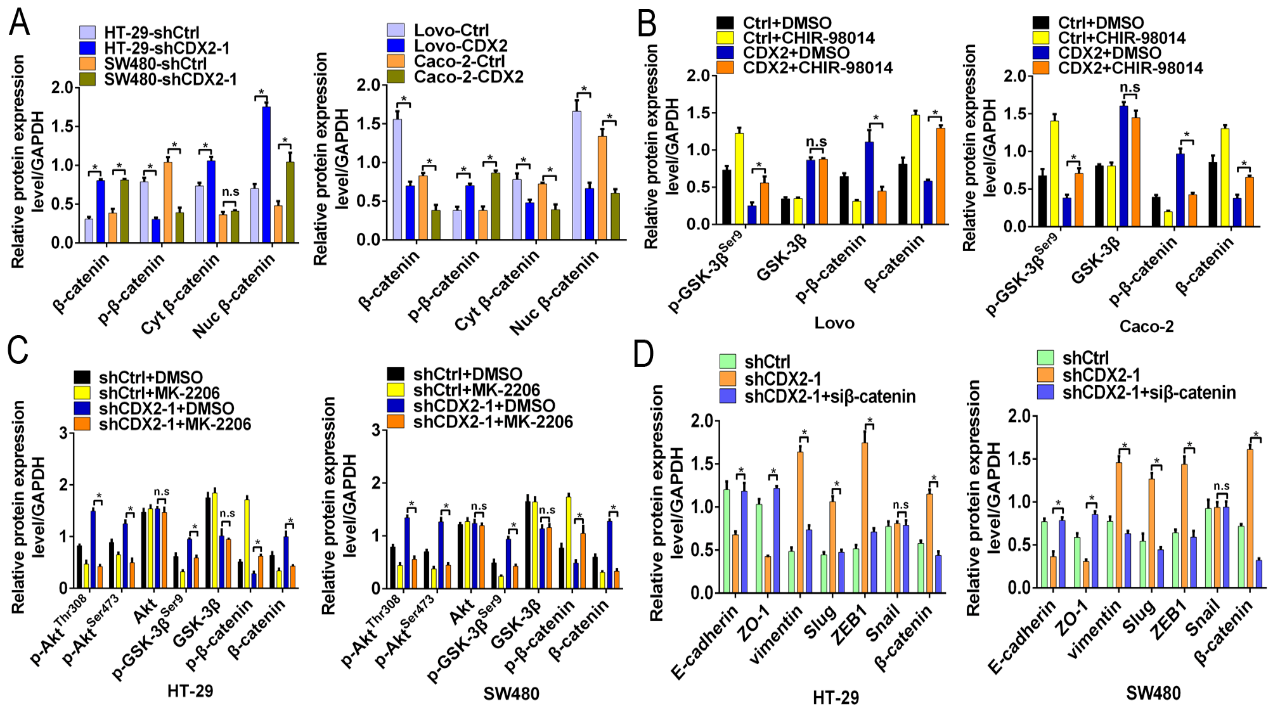


**Supplementary Fig. 7 CDX2 destabilizes β-catenin in CRC through PI3K/Akt/GSK-3β pathway.a** Quantitative analysis of nuclear β-catenin and total/phospho β-catenin protein in CDX2-knockdown and CDX2-overexpressing cells. **b** Quantitative analysis of GSK-3β/p-GSK-3β (Ser9) and β-catenin/p-β-catenin (Ser33/37/Thr41) proteins in CDX2-overexpressing cells treated with CHIR-98014. **c** Quantitative analysis of PI3K/Akt signaling-related proteins and β-catenin/p-β-catenin (Ser33/37/Thr41) proteins in CDX2-knockdown cells treated with MK-2206. **d** Quantitative analysis of EMT-related proteins in CDX2-knockdown cells transfected with siβ-catenin. All data are presented as the mean±SD from three independent experiments. **P* <0.05.

**Supplementary Fig. 8 CDX2 regulates stabilization of β-catenin in colon cancer cells**


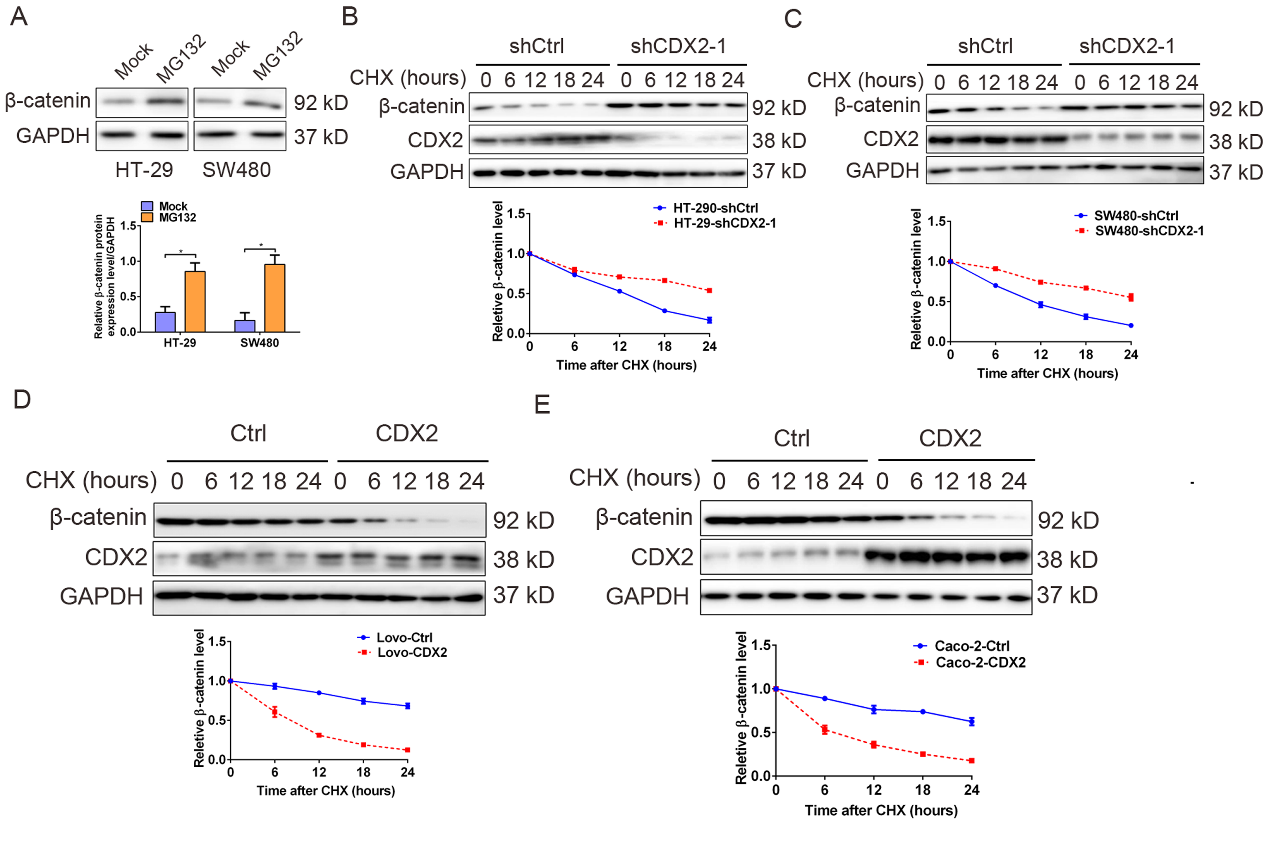


**Supplementary Fig. 8 CDX2 regulates stabilization of β-catenin in colon cancer cells. a** Western blotting analysis of β-catenin in HT-29 and SW480 cells treated with the proteasome inhibitor MG132. **b, c** Western blotting analysis of β-catenin in CDX2-knockdown HT-29 (**b**) and SW480 (**c**) treated with 50 μg/ml of CHX. The cells were harvested at the indicated times. Depletion of CDX2 [lengthen](javascript:;)ed the half-life of β-catenin protein. **d, e** Western blotting analysis of β-catenin in CDX2-overexpressing-Lovo (**d**) and Caco-2 (**e**) treated with 50 μg/ml of CHX. The cells were harvested at the indicated times. Enhanced CDX2 expression accelerated β-catenin degradation. All data are presented as the mean±SD from three independent experiments. **P* <0.05.

**Supplementary Fig. 9 CDX2 destabilizes β-catenin in CRC through PI3K/Akt/GSK-3β pathway**


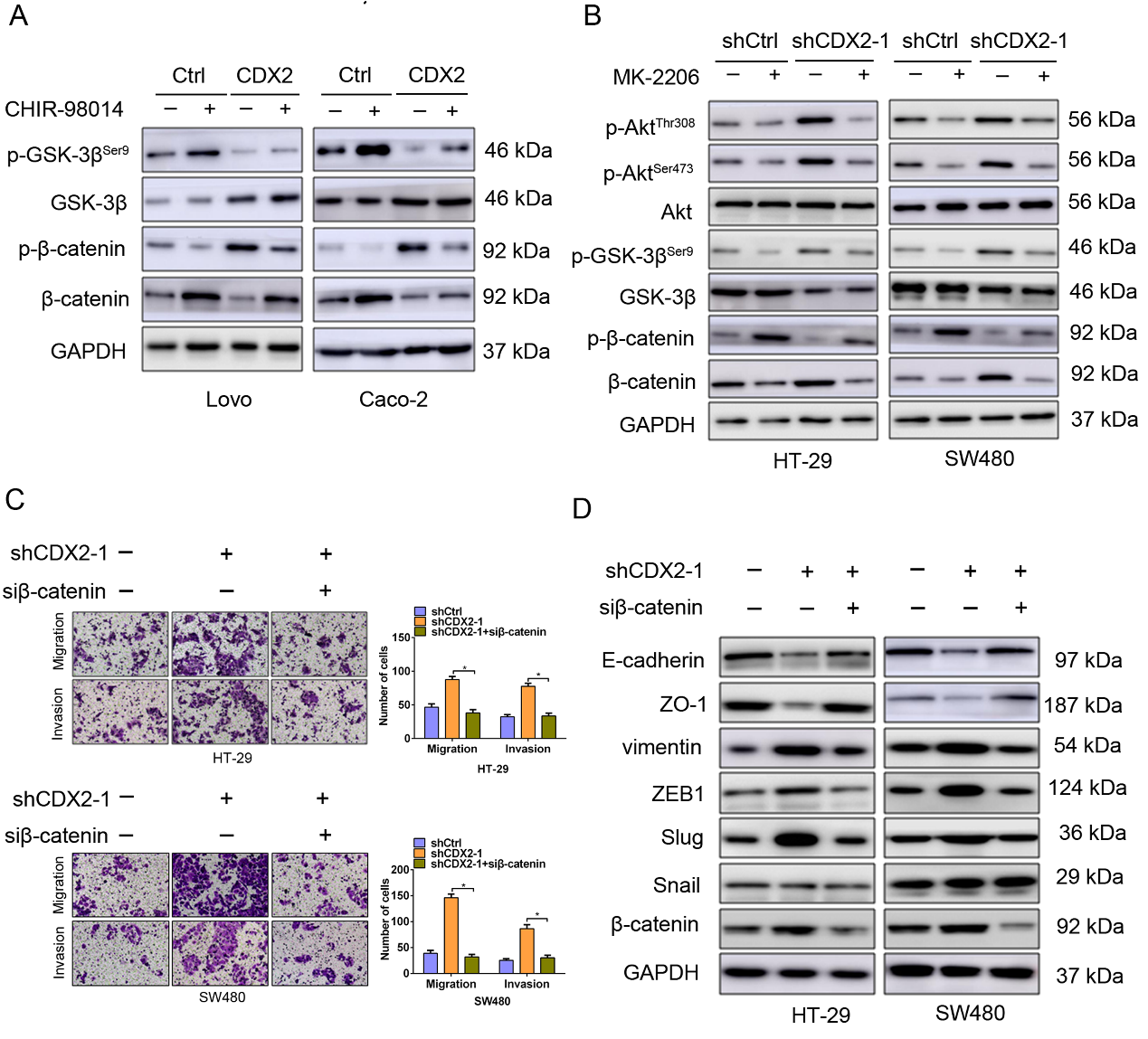


**Supplementary Fig. 9 CDX2 destabilizes β-catenin in CRC through PI3K/Akt/GSK-3β pathway**. **a** Western blotting bands for GSK-3β/p-GSK-3β (Ser9) and β-catenin/p-β-catenin (Ser33/37/Thr41) proteins in CDX2-overexpressing cells treated with CHIR-98014. **b** Western blotting bands for PI3K/Akt signaling-related proteins and β-catenin/p-β-catenin (Ser33/37/Thr41) proteins in CDX2-knockdown cells treated with MK-2206. **c** Transwell assays in CDX2-knockdown cells transfected with siβ-catenin. **d** Western blotting bands for EMT-related proteins in CDX2-knockdown cells transfected with siβ-catenin. All data are presented as the mean±SD from three independent experiments. **P* <0.05.

**Supplementary Fig. 10 CDX2 inhibits the activity of PI3K/Akt signaling by up-regulating PTEN expression**


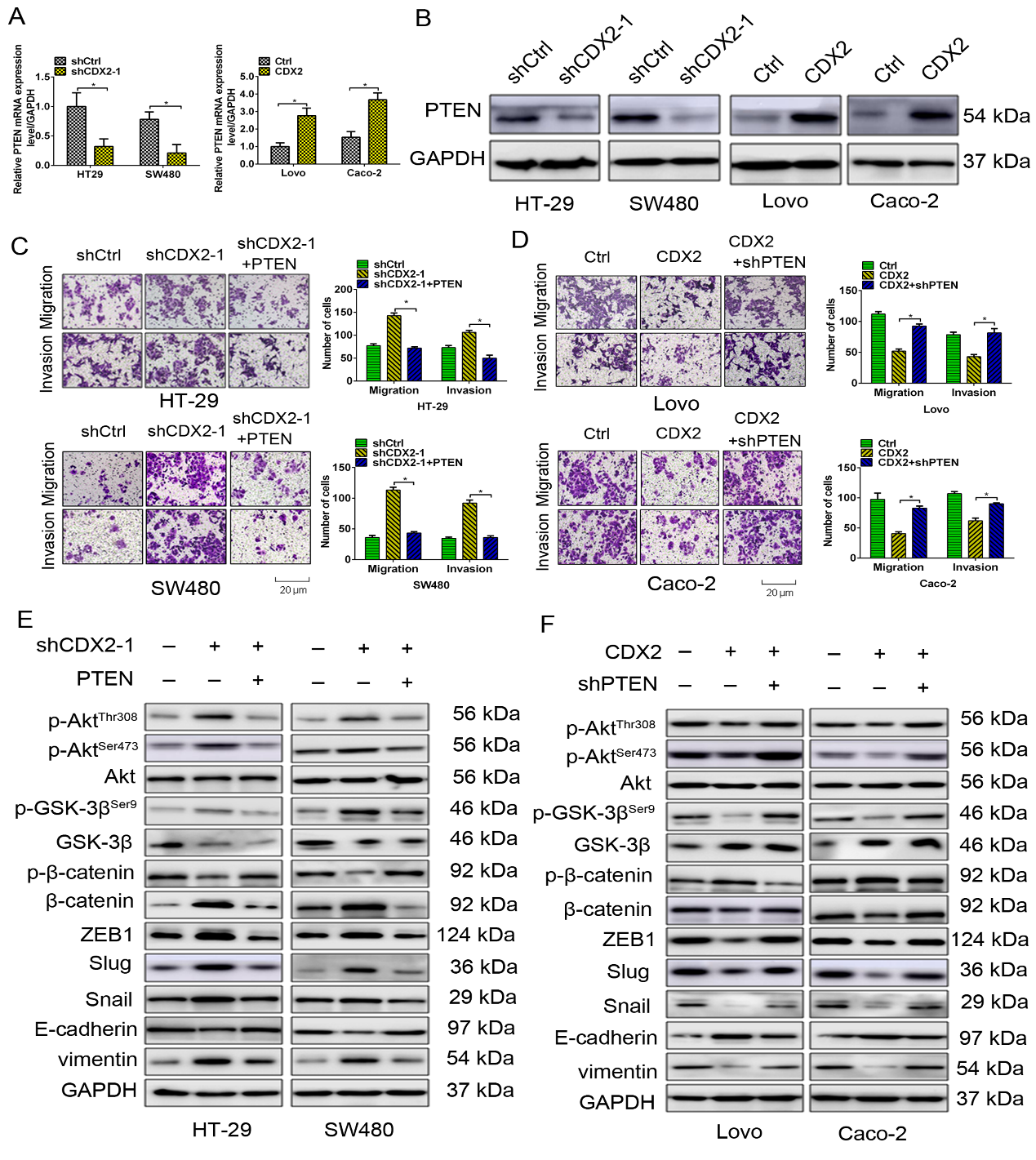


**Supplementary Fig. 10 CDX2 inhibits the activity of PI3K/Akt signaling by up-regulating PTEN expression. a, b** Real-time PCR (**a**) and western blotting analysis (**b**) of PTEN in CDX2-knockdown and CDX2-overexpressing cells. **c, d** Transwell assays in CDX2-knockdown (**c**) or CDX2-overexpressing cells (**d**) with PTEN overexpression or knockdown. **e, f** Western blotting bands for PI3K/Akt signaling-related proteins, β-catenin/p-β-catenin (Ser33/37/Thr41) and EMT-related proteins in CDX2-knockdown (**e**) and CDX2-overexpressing cells (**f**) with PTEN overexpression or knockdown. All data are presented as the mean±SD from three independent experiments. **P* <0.05.

**Supplementary Fig. 11 CDX2 inhibits the activity of PI3K/Akt signaling by up-regulating PTEN expression**


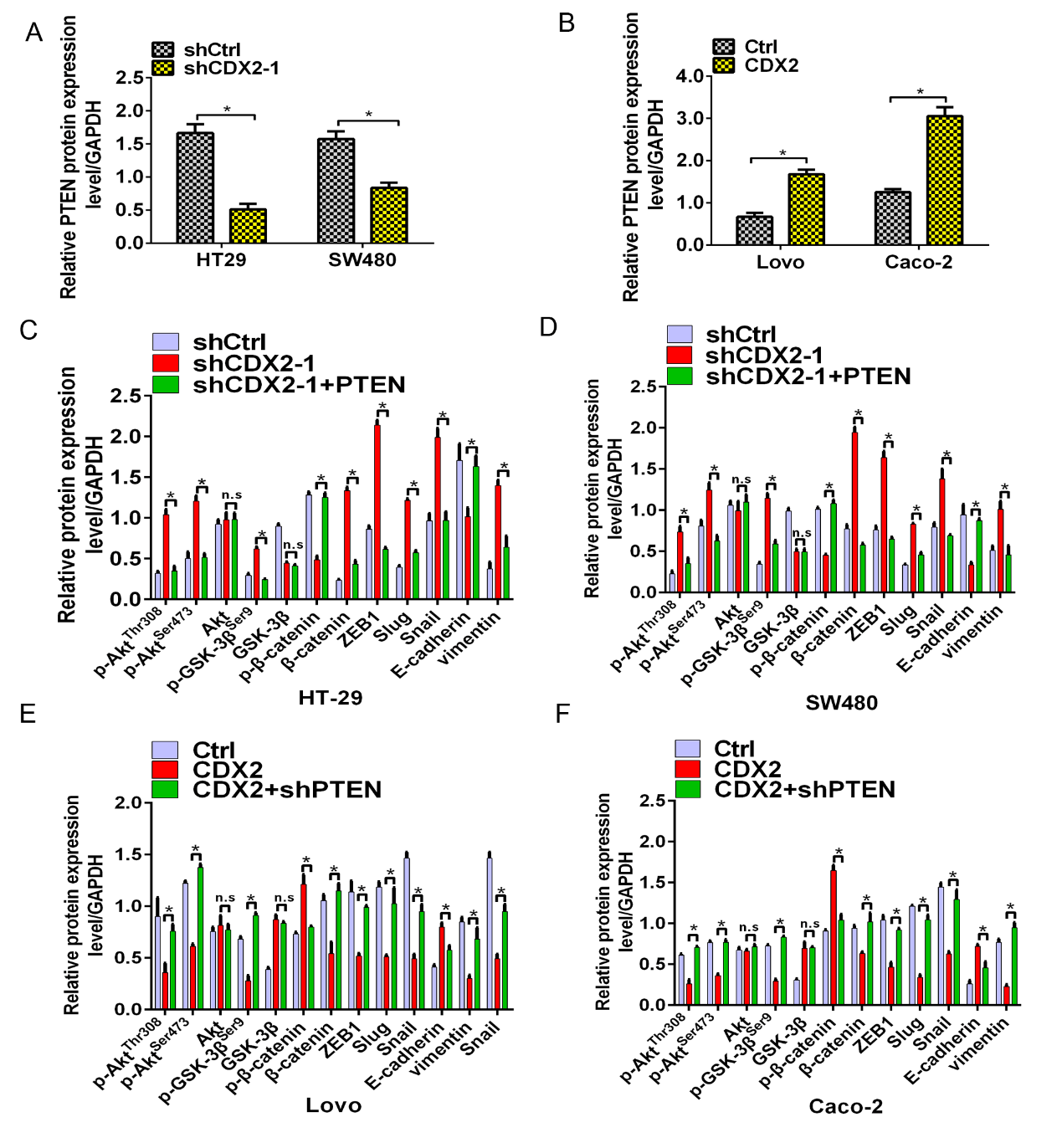


**Supplementary Fig. 11 CDX2 inhibits the activity of PI3K/Akt signaling by up-regulating PTEN expression. a, b** Quantitative analysis of PTEN protein in CDX2-knockdown (**a**) and CDX2-overexpressing (**b**) cells. **c, d** Quantitative analysis of PI3K/Akt signaling-related proteins, β-catenin/p-β-catenin (Ser33/37/Thr41) and EMT-related proteins in CDX2-knockdown HT-29 (**c**) and SW480 (**d**) cells with PTEN overexpression. **e, f** Quantitative analysis of PI3K/Akt signaling-related proteins, β-catenin/p-β-catenin (Ser33/37/Thr41) and EMT-related proteins in CDX2-overexpressing Lovo (**e**) and Caco-2 cells (**f**) with PTEN knockdown. All data are presented as the mean±SD from three independent experiments. **P* <0.05.

**Supplementary Fig. 12 Correlations among CDX2, PTEN, Snail, E-cadherin and vimentin expression levels in CRC tissues**


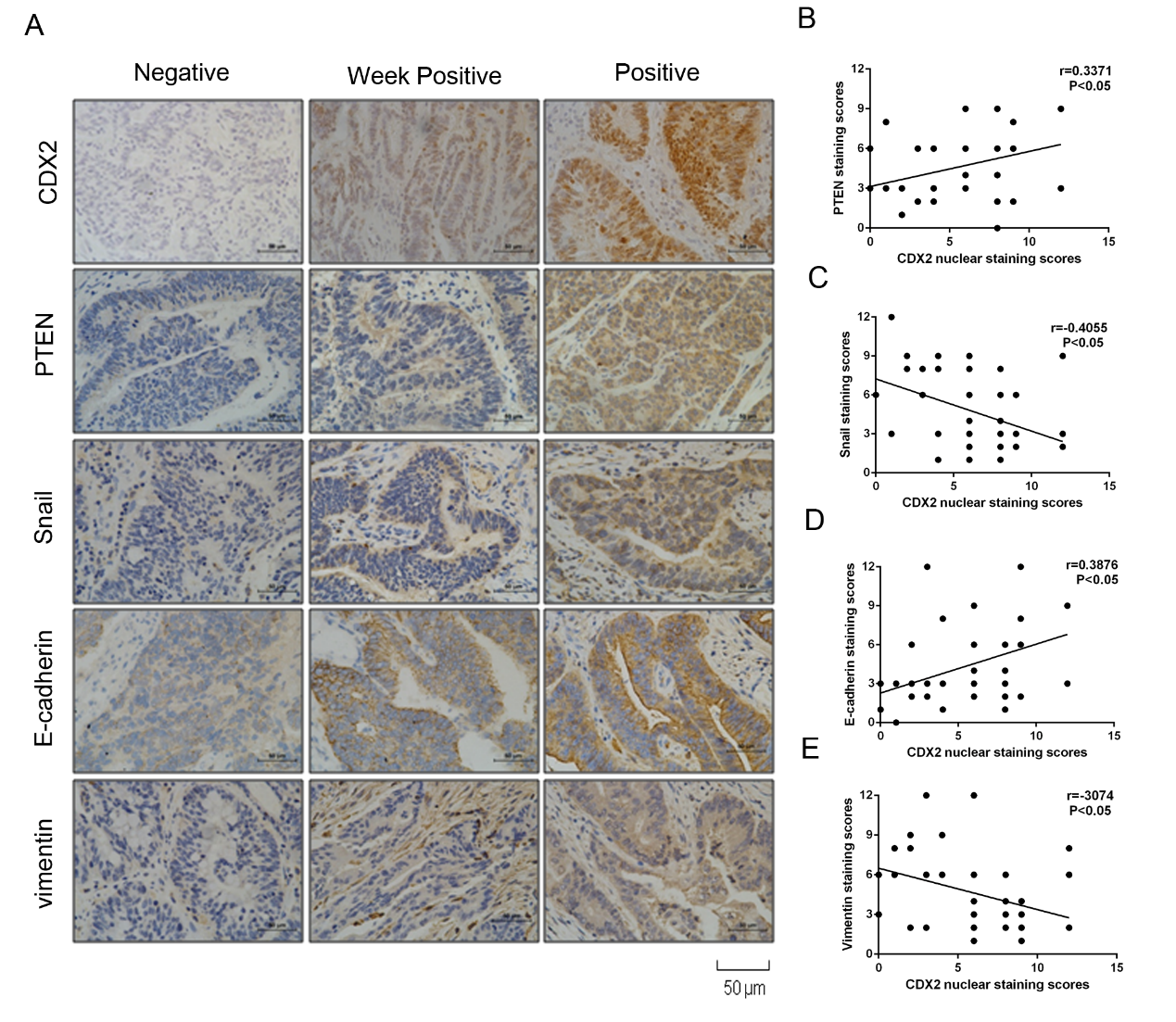


**Supplementary Fig. 12 Correlations among CDX2, PTEN, Snail, E-cadherin and vimentin expression levels in CRC tissues.** **a** CDX2, PTEN, Snail, E-cadherin and vimentin expression in CRC tissues using Immunohistochemistry (IHC) staining. **b, c, d, e** Correlations of CDX2 staining with PTEN (**b**, r=0.3371; *P*<0.05), Snail (**c**, r=-0.4055; *P*<0.05), E-cadherin (**d**, r=0.3878; *P*<0.05) and vimentin (**e**, r=-0.3074; *P*<0.05) levels. All data are presented as the mean±SD from three independent experiments.

Supplementary Table 1 Primer sequence

| Gene | Sequence |
| --- | --- |
| RT-PCR | |
| MMP-2 | F: 5’-ATGACATCAAGGGCATTCAGGAG-3’ |
|  | R: 5’-TCTGAGCGATGCCATCAAATACA -3’ |
| MMP-9 | F: 5’-TCCCAGACCTGGGCAGATTC -3’ |
|  | R: 5’-GCAAAGGCGTCGTCAATCAC-3’ |
| E-cadherin | F: 5’- TACACTGCCCAGGAGCCAGA-3’ |
|  | R: 5’-TGGCACCAGTGTCCGGATTA -3’ |
| ZO-1 | F: 5’-CAACATACAGTGACGCTTCACA-3’ |
|  | R: 5’-GACGTTTCCCCACTCTGAAAA-3’ |
| fibronectin | F: 5’-CCCCATTCCAGGACACTTCTG-3’ |
|  | R: 5’-GCCCACGGTAACAACCTCTT-3’ |
| vimentin | F: 5’-TGAGTACCGGAGACAGGTGCAG-3’ |
|  | R: 5’-TAGCAGCTTCAACGGCAAAGTTC-3’ |
| PTEN | F: 5’-CAAGATGATGTTTGAAACTATTCCAATG-3’ |
|  | R: 5’-CCTTTAGCTGGCAGACCACAA-3’ |
| GAPDH | F: 5’-TGCACCACCAACTGCTTAGC-3’ |
|  | R: 5’-GGCATGGACTGTGGTCATGAG-3’ |
| Luciferase Assays |  |
| F1(-912 bp **-** +207 bp) | F: 5’-CGGGGTACCGATGTGGCGGGACTCTTTAT-3’ |
| F2(-651 bp **-** +207 bp) | F: 5’-CGGGGTACC-GTTTCTCGCCTCCTCTTCGT-3’ |
| F3(-272 bp **-** +207 bp) | F: 5’-CGGGGTACCGCCGTTCGGAGGATTATT-3’ |
|  | R: 5’- GGAAGATCTTGACCTAGCAACCTGACCAG -3’ |
| ChIP-qPCR  P1(-912 bp **-** -757 bp) | F: 5’-GATGTGGCGGGACTCTTTAT-3’ |
|  | F: 5’-CTCATCTCCCTCGCCTGAG-3’ |
| P2(-425 bp- -254) | F: 5’-GAACGCCGGAGAGTTGGT-3’ |
|  | F: 5’-GAATAATCCTCCGAACGGC-3’ |
| P3(+44 bp- +159 bp) | F: 5’-GATATCAAGAGGATGGATTCGACT-3’ |
|  | F: 5’-GGACATTTTCGCATCCGTCT-3’ |
| P4(+140 bp- +207) | F: 5’-AGACGGATGCGAAAATGTCC-3’ |
|  | F: 5’-TGACCTAGCAACCTGACCAG-3’ |
| 3’-UTR | F: 5’-CACTCCACTTTCTGTAAAGGCAATG-3’ |
|  | F: 5’-TGTCCCAAAGTATCAGTCTAAATGG-3’ |

Supplementary Table 2 Antibodies

| Antibodies | Source | | Identifier |
| --- | --- | --- | --- |
| GAPDH | | Santa Cruz | Cat#sc-47724 |
| vimentin | | Abcam | Cat#ab92547 |
| MMP9 | | Abcam | Cat#ab76003 |
| Snail | | Abcam | Cat#ab53519 |
| Slug | | Abcam | Cat#ab27568 |
| Twist | | Abcam | Cat#ab50581 |
| fibronectin | | Abcam | Cat#ab32419 |
| AKT | | Abcam | Cat#ab8805 |
| p-AKT^Thr308^ | | Abcam | Cat#ab38449 |
| p-AKT^Ser473^ | | Abcam | Cat#ab81283 |
| CDX2 | | Cell Signaling Technology | Cat#12306 |
| Erk1/2 | | Cell Signaling Technology | Cat#4695 |
| p-Erk1/2^Thr202/Tyr204^ | | Cell Signaling Technology | Cat#8544 |
| GSK-3β | | Cell Signaling Technology | Cat#12456 |
| GSK-3β ^Ser9^ | | Cell Signaling Technology | Cat#5558 |
| E-cadherin | | Cell Signaling Technology | Cat#14472 |
| ZEB1 | | Cell Signaling Technology | Cat#55506 |
| ZEB2 | | Cell Signaling Technology | Cat#18583 |
| ZO-1 | | Cell Signaling Technology | Cat#13663 |
| β-catenin | | Cell Signaling Technology | Cat#8480 |
| p-β-catenin^Ser33/37/Thr41^ | | Cell Signaling Technology | Cat#9561 |
| Histone H3 | | Cell Signaling Technology | Cat#4499 |

Supplementary Table 3 Correlation between the factors and clinicopathologic

characteristics in CRC

| Clinicopathologic indexes | CDX2 | | χ² | P value |
| --- | --- | --- | --- | --- |
|  | Negative | Positive |  |  |
| Gender |  |  | 0.082 | 0.775 |
| Male | 21 | 75 |  |  |
| Female | 13 | 52 |  |  |
| Age in diagnosis(years) |  |  | 0.072 | 0.789 |
| ≤55 | 12 | 48 |  |  |
| >55 | 22 | 79 |  |  |
| Differentiation |  |  |  | 0.066^#^ |
| High/Moderate | 27 | 116 |  |  |
| Low/Undifferentiated | 7 | 11 |  |  |
| Tumor size |  |  | 0.362 | 0.548 |
| ≤5cm | 22 | 89 |  |  |
| >5cm | 12 | 38 |  |  |
| T stages |  |  | 2.017 | 0.156 |
| T1-T2 | 4 | 29 |  |  |
| T3-T4 | 30 | 98 |  |  |
| Lymphatic metastasis |  |  | 30.154 | <0.001* |
| No | 10 | 100 |  |  |
| Yes | 24 | 27 |  |  |
| M stages |  |  |  | 0.002^#,^* |
| No | 25 | 119 |  |  |
| Yes | 9 | 8 |  |  |
| TNM stages |  |  | 30.154 | <0.001* |
| Ⅰ+Ⅱ | 10 | 100 |  |  |
| Ⅲ+Ⅳ | 24 | 27 |  |  |
| Location |  |  | 2.024 | 0.155 |
| Right | 21 | 61 |  |  |
| Left | 13 | 66 |  |  |

#: Fisher’s test

**P*<0.05

Supplementary Table 4 Cox proportional Hazard regression analysis of patients’ overall survival

| Varibles | Univariable | | | | | |  | Multivariable | | | |
| --- | --- | --- | --- | --- | --- | --- | --- | --- | --- | --- | --- |
|  |  | | 95.0% CI | | |  |  |  | 95.0% CI | |  |
|  | HR | | lower | | upper | P |  | HR | lower | Upper | P |
| Age  (>55 vs ≤55 ) | 1.171 | 0.749 | | 1.830 | | 0.488 |  |  |  |  |  |
| Gender  (Male vs Female ) | 0.853 | 0.553 | | 1.314 | | 0.470 |  |  |  |  |  |
| Differentiation  (Low/Undifferentiated vs High/Moderate ) | 4.413 | 2.526 | | 7.708 | | <0.001* |  | 0.996 | 0.518 | 1.915 | 0.990 |
| Tumor size  (>5cm vs ≤5cm) | 2.324 | 1.499 | | 3.603 | | <0.001* |  | 1.626 | 0.979 | 2.700 | 0.060 |
| T stages  (T3-T4 vs T1-T2) | 3.006 | 1.503 | | 6.011 | | 0.002* |  | 1.813 | 0.883 | 3.723 | 0.105 |
| Lymphatic metastasis  (Yes vs No) | 6.528 | 4.186 | | 10.180 | | <0.001* |  | 6.212 | 3.572 | 10.801 | <0.001* |
| M stages  (Yes vs No) | 19.209 | 9.611 | | 38.391 | | <0.001* |  | 6.506 | 2.788 | 15.237 | <0.001* |
| TNM stages  (Ⅲ+ⅣvsⅠ+Ⅱ) | 6.528 | 4.186 | | 10.180 | | <0.001* |  | NA | NA | NA | NA |
| Location  （Right vs Left） | 1.359 | 0.883 | | 2.091 | | 0.163 |  |  |  |  |  |
| CDX2 expression  (High vs Low) | 0.598 | 0.367 | | 0.973 | | 0.038* |  | 1.961 | 1.112 | 3.459 | 0.020* |

NA: Stage=N1

**P*<0.05

Supplementary Table 5 Cox proportional Hazard regression analysis of patients’ recurrence-free survival

| Varibles | Univariable | | | | |  | Multivariable | | | |
| --- | --- | --- | --- | --- | --- | --- | --- | --- | --- | --- |
|  |  | 95.0% CI | | |  |  |  | 95.0% CI | |  |
|  | HR | lower | upper | P | |  | HR | lower | Upper | P |
| Age  (>55 vs ≤55 ) | 1.214 | 0.762 | 1.936 | 0.415 | |  |  |  |  |  |
| Gender  (Male vs Female ) | 1.031 | 0.655 | 1.623 | 0.894 | |  |  |  |  |  |
| Differentiation  (Low/Undifferentiated vs High/Moderate ) | 4.066 | 2.297 | 7.198 | <0.001* | |  | 1.248 | 0.660 | 2.362 | 0.496 |
| Tumor size  (>5cm vs ≤5cm) | 2.104 | 1.338 | 3.310 | 0.001* | |  | 1.263 | 0.733 | 2.178 | 0.401 |
| T stages  (T3-T4 vs T1-T2) | 2.996 | 1.441 | 6.231 | 0.003* | |  | 1.992 | 0.939 | 4.225 | 0.072 |
| Lymphatic metastasis  (Yes vs No) | 6.218 | 3.912 | 9.882 | <0.001* | |  | 4.346 | 2.462 | 7.671 | <0.001* |
| M stages  (Yes vs No) | 14.025 | 7.355 | 26.743 | <0.001* | |  | 4.073 | 1.880 | 8.824 | <0.001* |
| TNM stages  (Ⅲ+ⅣvsⅠ+Ⅱ) | 6.218 | 3.912 | 9.822 | <0.001* | |  | NA | NA | NA | NA |
| Location  （Right vs Left） | 1.452 | 0.927 | 2.276 | 0.104 | |  |  |  |  |  |
| CDX2 expression  (High vs Low) | 0.458 | 0.283 | 0.741 | 0.001* | |  | 1.058 | 0.616 | 1.820 | 0.837 |

NA: Stage=N1

**P*<0.05
